# Performance investigation of state-of-the-art metaheuristic techniques for parameter extraction of solar cells/module

**DOI:** 10.1038/s41598-023-37824-4

**Published:** 2023-07-10

**Authors:** Abhishek Sharma, Abhinav Sharma, Moshe Averbukh, Vibhu Jately, Shailendra Rajput, Brian Azzopardi, Wei Hong Lim

**Affiliations:** 1 Department of Computer Science and Engineering, Graphic Era Deemed to Be University, Dehradun, 248002 India; 2 Department of Electrical and Electronics Engineering, University of Petroleum and Energy Studies, Dehradun, 248007 India; 3 Department of Electrical and Electronics Engineering, Ariel University, 40700 Ariel, Israel; 4 College of Engineering, Xi’an International University, Xi’an, 710077 China; 5 MCAST Energy Research Group (MCAST Energy), Institute of Engineering and Transport, Malta College of Arts, Science and Technology (MCAST), Triq Kordin, Paola, PLA 9032 Malta; 6 Faculty of Engineering, Technology and Built Environment, UCSI University, 56000 Cheras, Kuala Lumpur, Malaysia; 7 The Foundation for Innovation and Research – Malta, 65 Design Centre Level 2, Tower Road, BKR 4012 Birkirkara, Malta

**Keywords:** Electrical and electronic engineering, Renewable energy, Computer science

## Abstract

One of the greatest challenges for widespread utilization of solar energy is the low conversion efficiency, motivating the needs of developing more innovative approaches to improve the design of solar energy conversion equipment. Solar cell is the fundamental component of a photovoltaic (PV) system. Solar cell’s precise modelling and estimation of its parameters are of paramount importance for the simulation, design, and control of PV system to achieve optimal performances. It is nontrivial to estimate the unknown parameters of solar cell due to the nonlinearity and multimodality of search space. Conventional optimization methods tend to suffer from numerous drawbacks such as a tendency to be trapped in some local optima when solving this challenging problem. This paper aims to investigate the performance of eight state-of-the-art metaheuristic algorithms (MAs) to solve the solar cell parameter estimation problem on four case studies constituting of four different types of PV systems: R.T.C. France solar cell, LSM20 PV module, Solarex MSX-60 PV module, and SS2018P PV module. These four cell/modules are built using different technologies. The simulation results clearly indicate that the Coot-Bird Optimization technique obtains the minimum RMSE values of 1.0264E-05 and 1.8694E−03 for the R.T.C. France solar cell and the LSM20 PV module, respectively, while the wild horse optimizer outperforms in the case of the Solarex MSX-60 and SS2018 PV modules and gives the lowest value of RMSE as 2.6961E−03 and 4.7571E−05, respectively. Furthermore, the performances of all eight selected MAs are assessed by employing two non-parametric tests known as Friedman ranking and Wilcoxon rank-sum test. A full description is also provided, enabling the readers to understand the capability of each selected MA in improving the solar cell modelling that can enhance its energy conversion efficiency. Referring to the results obtained, some thoughts and suggestions for further improvements are provided in the conclusion section.

## Introduction

Various environmental issues such as air pollution, water pollution and global warming have recently become the main concerns of the scientific community, the policy makers and the public at large. This increased awareness has culminated in United Nation’s Sustainable Development Goals (SDGs)^1^. Rapid technological advancement due to the spread of Industrial Revolution 4.0 (IR 4.0) in newer regions of the world and unchecked population growth have been the two major factors responsible for a manifold increase of energy consumption. Most of this increased demand has been met from the conventional power plants fired with fossil fuels. These fuels offered good efficiency, as well as the ease of transportation. Access to these fuels was easy too. Nevertheless, the widespread utilization of these fossil fuels in power and utility industry have caused irreversible adverse effects on the environment resulting in climate change, global warming, air pollution and water pollution; overall making reliance on these fossil fuels unsustainable. The detrimental side effects brought by these environmental hazards can trigger additional issues, especially those related to the human health and morbidity. Other issue with the fossil fuels is their diminishing stocks on our planet. These stocks are estimated to last only for a century or two depending on the rate at which these are extracted from the earth. To address this problem of diminishing stocks and to obviate the undesirable effects of the fossil fuels, there is a growing trend of exploring alternative energy sources that are both renewable and more environmental-friendly in nature, such as wind, tidal, biomass, solar, water and geothermal energy to satisfy the ever-growing energy demands.

Among all aforementioned renewable energy sources, solar energy is envisioned as a promising alternative of conventional fossil fuels for power generation. A typical PV system used to directly convert the solar energy into electricity consists of fundamental components known as solar cell, i.e., a semiconductor diode with P–N junction exposed to the light. A PV module is formed by connecting some solar cells in series, whereas a PV panel is constructed by connecting several PV modules in series and parallel. Meanwhile, a PV array may comprise single or multiple PV modules. Finally, a complete PV system involves PV arrays, DC to DC boost converter, maximum power point tracking systems and inverters (only for grid connected PV systems)^2^. As compared to conventional fossil fuels and other renewable energy sources, solar energy has more desirable characteristics such as the omnipresent source of electricity, lower operational costs, ease of installation, scalability and noise-free generation^3^. It is also noteworthy that the production cost of solar cells has decreased significantly from $76.67 per watt in 1977 to $0.37 per watt in 2017^4^. These competitive advantages of solar energy have attracted substantial amounts of financial incentives from both public and private sectors to promote its wide range of applications (e.g., electric power generation, water heating, and water pumping), enabling it to be the third largest renewable energy source in global after hydropower and wind energy^5^. In 2010, the worldwide installed solar capacity was 72.04 GW and it has increased by almost ten times to 707.50 GW during 2020^5^. It was anticipated that the installed solar capacity can reach 1 TW by the end of 2021 based on the rapid growing trend of solar energy^6^. Despite the benefits offered against other renewable energy sources; solar energy is not without its limitations. One of the major constraints is the high initial cost required for the installation of PV system to generate electricity. Maintenance costs are also incurred periodically to repair or replace the degraded PV modules that normally remain exposed to outdoor environments during their operations. It is also notable that the power generated by a PV system is not controllable because it varies with various environmental factors such as irradiation and temperature. Extensive amounts of researches have been carried out to identify and resolve the issues related to PV system from different perspectives in order to enhance its power generation efficiency with lower overall costs.

The appropriate modelling of solar cells or PV modules are imperative to analyse and evaluate the actual behaviour of PV systems under diverse operating conditions. An accurate and efficient PV model can be utilized for the simulation, design, control, and optimisation of PV system (e.g., maximum generated output power). Most often, precise modelling of PV cells involves the proper selection of modelling technique and good estimation of model parameters. The behaviour of solar cell is represented using current–voltage (I–V) characteristics, where the latter are determined by solving the partial differential equations (PDEs) used for describing physical phenomena of elementary charges (holes and electrons) movement in the matter of a semiconductor and in the vicinity of potential barrier. To tackle high complexity issue encountered in solving PDEs, more computationally efficient equivalent circuit models consisting of diode and resistors are derived with Kirchhoff equations and used to emulate the electrical behaviour of PV cell^7^. Some notable equivalent circuit models used to represent solar cell include the single diode model (SDM)^8^, double diode model (DDM)^9^ and triple diode model (TDM) with five, seven and nine unknown parameters, respectively. The accuracy and complexity of these equivalent circuit models tend to increase along with the number of unknown parameters. Appropriate circuit models need to be carefully selected for PV applications by considering the proper trade-offs between their accuracy and complexity. For instance, both of the SDM and DDM are commonly used for domestic purpose, whereas the TDM^10^ is more feasible for industrial application given its capability to avoid the faulty issues caused by recombination process.

Apart from the appropriate selection of modelling technique for solar cell, the accurate estimation of its model parameters is also crucial to correctly simulate the behaviour of solar cell under different operating conditions in order to ensure optimal performance of PV system. Nevertheless, it is nontrivial to estimate the solar cell model parameter accurately due to the nonlinear characteristic of I–V curve. The exact values of these solar cell model parameters are often not available due to their tendency to change with different operating conditions and influence from other factors such as the physical structures, types and aging effect of solar cell. Motivated by these challenging issues, a large number of parameter estimation techniques has been proposed by researchers to identify the unknown parameters of solar cell models accurately and efficiently^11^. A popular approach used to determine the best combination of these unknown model parameters is to represent the parameter estimation of solar cell or PV model as an optimization problem and solve the objective function derived based on nonlinear I–V curve of solar cell or PV module^12^. Traditional optimization methods with deterministic nature such as Newton Raphson^13^, Gauss–Seidel method^14^ and Lambert-W functions^15^ tend to be trapped in local optima and produce poor quality solutions due to the complex search space of PV model parameter estimation problem with nonlinear and multimodal properties. In addition, the performances of these traditional optimization methods are also highly dependent on the initial solutions produced as well as the continuity, convexity and differentiability of given objective functions, thereby restricting their practical applications in real-world scenario^16^.

**Figure 1:**
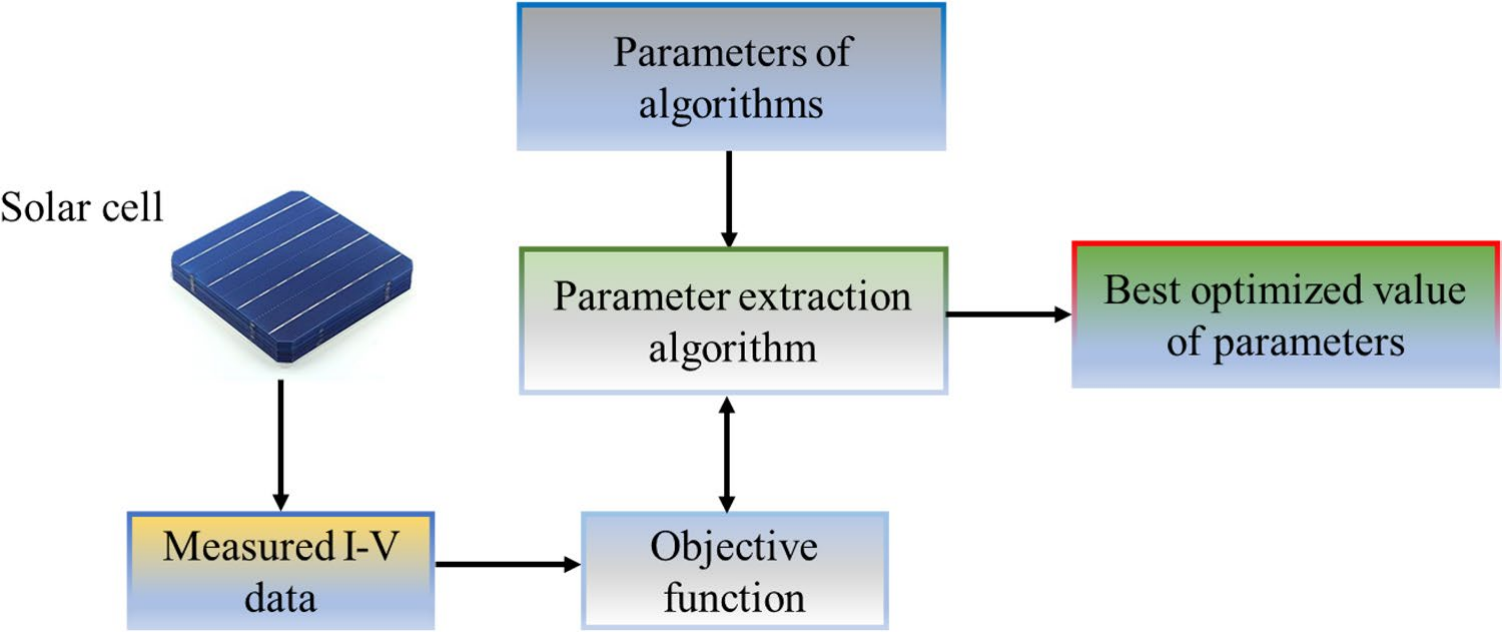
Process flow diagram of parameter estimation of solar cell.

Motivated by the drawbacks of traditional optimization methods, there are growing trends of employing metaheuristic algorithms (MAs) to tackle PV model parameter estimation problems represented with SDM, DDM or TDM. Depending on the source of inspirations used for emulating search mechanisms, existing MAs can be broadly classified into four types^17^, namely evolutionary algorithms, swarm intelligence algorithms, physics-based algorithms and human-based algorithms. In contrast to the traditional optimization methods, these MAs offer more competitive advantages in extracting the optimal model parameters of solar cell or PV module given their better global search capability, lower sensitivity on the initial solutions and lesser dependence on gradient information of objective function. The process flow diagram of using MAs for parameter assessment of solar cell or PV module is depicted in Fig. 1.

This process is followed in most studies. Reference to some interesting recent works follow. The authors proposed improved electromagnetism-like algorithm for parameter extraction of polycrystalline, monocrystalline and thin film PV modules^18^. The quick convergence rate of the algorithm is highly dependent on the accuracy of the measured data and is more suitable for DDM and TDM. In^19^, the authors proposed tree seed algorithm for parameter estimation of PV module. The proposed algorithm is robust and has a high accuracy, its performance remains in doubt under variation in irradiance. In another study^20^, the authors proposed an improved queuing search optimization (QSO) algorithm dependent on the differential evolution technique (DE) and bound-constraint amendment procedure for parameter estimation of PV modules. The proposed technique applied DE algorithm to each solution generated by the QSO algorithm in order to increase population diversity. In^21^, the authors proposed an improved Moth Flame Algorithm (MFO) with local escape operators. The local escape operator technique improves the MFO algorithm’s exploration efficiency and the diversity of the population. The researchers suggested dI/dV-assisted deterministic method to extract the parameters using SDM of PV cell. The study suggests that not only the number of iteration steps but also the single-step computation complexity should be taken into account for comparing deterministic optimization algorithms^22^. The authors proposed Harris Hawks optimization algorithm to extract the parameters of PV modules using TDM. The study used unimodal, multimodal and fixed-dimension benchmark functions to verify the quality and efficiency of the proposed method^23^. In another study the authors investigated a decent basis for proper investigation and the implementation of atomic orbital search algorithm to estimate the PV parameters using SDM, DDM and TDM. The proposed method exhibited the lowest root mean square error among the compared metaheuristic techniques^24^. In^25^, the researchers suggested optimization of PV module parameters using a modified quasi-oppositional logistic chaotic rao-1 (QOLCR) algorithm. The work indicates that the QOLCR approach converges faster than the basic Rao-1 algorithm and its other variants.

Meanwhile, Table 1 summarizes the existing review papers related to parameter extraction of solar cell and PV module in terms of their author names, year of publication, techniques reviewed and types of review. Although MAs generally exhibited better performance than the traditional optimization methods when solving PV model parameter estimation problems, some MAs might have slow convergence speed to locate global optimum or tend to produce inconsistent results in different trials due to their stochastic characteristic. Extensive research efforts are still being put to design more robust parameter identification approaches that can solve PV model parameter estimation problem. For example, thermo-economic optimization of flat-plate solar collector systems, optimum allocation of distributed generation and optimum power flow to minimize active power losses remain unexplored^26–28^. Motivated by No Free Lunch (NFL) Theorem^29^, numerous new MAs were designed to solve the global optimization problems but their performances were only evaluated based on standard benchmark functions. It is crucial to conduct further investigation to validate the practicability of these emerging MAs in real-world applications. Hence, the proposed work evaluates the performance of eight state-of-the-art MAs in estimating the PV cell parameters on four case studies using four different types of PV cells/modules under wide range of irradiance and temperature levels. On the basis of the literature review the following research gaps have been identified.

- There is a need of comparative study which analyses the performance of state-of-the-art MAs in estimating PV model parameters.
- There exists a research gap in evaluating the performance of MAs in identifying parameters of different PV cell technologies under wide range of irradiance and temperature levels.
- There is a need to thoroughly investigate the performance of recently develop MAs using statistical techniques to demonstrate their robustness.

**Table 1:** Comparison of previously published review papers for parameter assessment of solar cell/PV module.

Authors	Techniques reviewed	Type of review	Remarks
Nayak et al.^30^	VIM, modified NR method, NLSA	Simulation	The accuracy of MAs depends on tolerance band and initial conditions Comparison is carried out based on execution time
Jordehi^31^	ABC, Penalty-based DE, Improved JADE, PSO, IGHS, BMO, modified TLBO, COA, SA, AIS	Theoretical	Measurement noise result is an important aspect which leads to inaccuracy in estimating the model parameters Tuning of the control parameters of metaheuristics is imperative
Abbassi et al. ^32^	DE, RADE, PDE, IADE, ABCO, AGA, APSO, BBO-M, BMO, CPSO, DEIM, HS, IGHS, GGHS, IBCPSO, ADE, LS, NR, PSA, SBMOA, GOTLBO, STLBO, TVIWAC-PSO, ABSO, AIS, ANN, BBO, BFA, BPFPA, GA, IGHS, IPSO, LM, MPCOA, PS, RADE, SA, TLBO, VC-PSO, NMS	Theoretical	A multitude of objective functions should be compared to efficiently select most appropriate parameters that describe the I-V characteristics of PV cell CPU execution time and the convergence rate are important attributes in identifying the performance of the optimization methods
Khursheed et al. ^33^	Numerical Approach, Lambert W-function, Explicit model, HSA, ANN, TLO, SBMOA, FWA, PSO, GA, BP-FPA, MSSO, ERWCA, IC-WOA, NMS-ABC, HBPFPA, TLABCA, GCPSO-NRM	Theoretical	Aging and damages due to weather conditions is vital to develop accurate models and obtain realistic results The performance of recently developed methods should be tested on new PV technologies such as organic and multi-junction cells
Oliva et al.^34^	GA, DE, HS, SA, PSO, CSO, ABC, WOA, GSA, FPA, SCE, WDO	Theoretical	WOA with small modifications exhibited the best performance Combination of MH with other approaches will improve the exploration thereby increasing the robustness and convergence rate of the method
Yang et al. ^35^	GA, DE, ABSO, ABC, WOA, IALO, CS, BMO, FPA, GWO, BFA, AIS, SSA, PSO, MSPCOA, SA, FWA, WDO, ERWCA, LMCOA, HS, TLO, ICA, MLBSA, PSA, SCE,	Theoretical	SA is not influenced by the change in irradiance level The accuracy of DDM is slightly higher than SDM based on the RMSE and MAE
Abdulrazzaq et al.^36^	SDM implicit – PSO, SDM explicit – PSO, LSM – PSO, LSM – Newton	Simulation	NR method provides the best results for the mono-crystalline PV cell PSO exhibited the longest average convergence time because of the implicit nature of the Lambert W-function
Venkateswari et al.^37^	Mutant PSO, performance-guided Jaya, FPA, GOFPANM, DE, BFA	Simulation	In monocrystalline cell FPA and SSA for SDM and DDM exhibits the best performance, respectively In thin-film cell PSO and BPFPA for SDM and DDM showed best performance, respectively

This article aims to analyse the performances of eight recently developed MAs for solving different case studies of PV model parameter estimation problems, particularly in terms of their accuracy, reliability, convergence speed and computational complexity. These eight selected MAs include Spotted Hyena Optimizer (SHO)^38^, Sooty Tern Optimization (STO)^39^, Aquila Optimization (AO)^40^, Harris Hawks Optimization (HHO)^41^, Wild Horse Optimization (WHO)^42^, Arithmetic Optimization Algorithm (AOA)^43^, Atom Search Optimization (ASO)^44^ and Coot Bird Optimization (CBO)^45^. The main contributions of this paper are summarized as follows:

- A detailed comparative study of recently developed MAs for parameter estimation of solar PV modules.
- A qualitative and quantitative analysis to evaluate the performance of state-of-the-art MAs for PV module parameter estimation based on key performance indices such as root mean square error (RMSE), computational complexity, current–voltage (I–V) characteristic curves, power-voltage (P–V) characteristic curves, and rate of convergence.
- An exhaustive statistical analysis using Friedman and Wilcoxon test to validate the robustness of the MAs.
- The performance evaluation of eight MAs for PV parameter estimation for four different solar PV modules on the basis of manufacturing technology, modelling of solar cells and environmental factors (i.e., temperature and irradiance levels).

The remaining sections in this paper are organized as follows: The mathematical modelling for the equivalent circuit of a solar cell is explained in Section “Formulation of solar cell/module parameter estimation problem”. Section “Estimation of solar cell/module” introduces the basic concepts and search mechanisms of all eight selected MAs. A comprehensive performance evaluation of eight MAs in solving four case studies of solar cell or PV module parameter estimation problem are discussed and summarized in Section “Results and discussion”. Finally, Section “Conclusion” delivers the conclusive remarks.

## Formulation of solar cell/module parameter estimation problem

### Equivalent circuit of SDM

**Figure 2:**
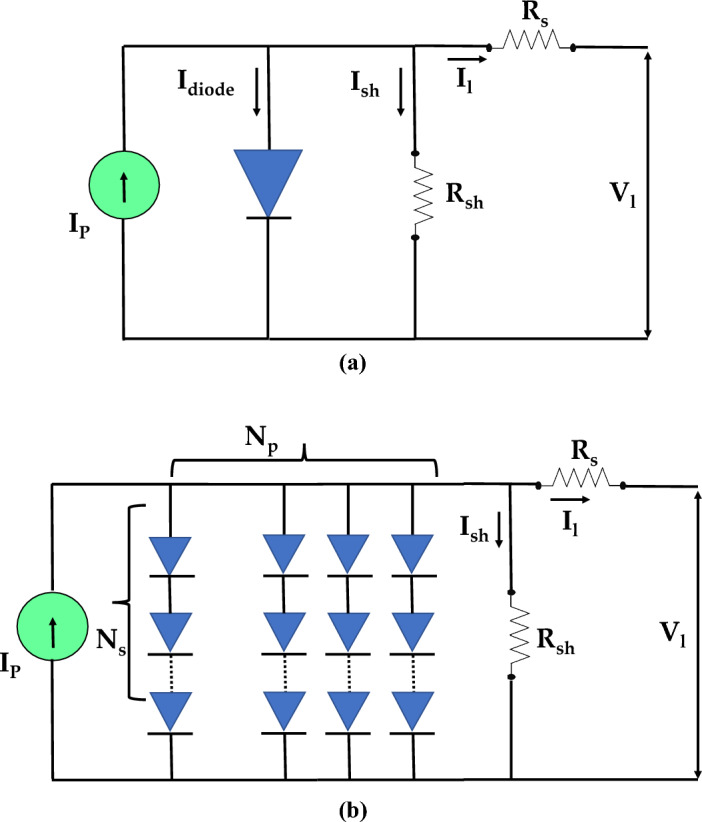
Equivalent circuit of SDM used to represent (**a**) solar cell (**b**) PV panel.

Figure 2 illustrates the equivalent circuit of SDM commonly used to represent a standard solar cell. SDM is chosen because of its simpler control topology, minimal circuit complexity, and the ease of hardware execution. A diode is connected in parallel with the photogenerated current source to define the non-physical factor for diode ideality in p–n junction. Meanwhile, the shunt resistor (*R*_*sh*_) and series resistor (*R*_*s*_) are presented to consider the ohmic losses due to carrier recombination and metallic junction, respectively. The electrical behaviour of a solar cell can be expressed by calculating its output current ${I}_{l}$ as shown in Eq. (1)^46^:

1 $${I}_{l}={I}_{p}-{I}_{diode}-{I}_{sh}$$

where *I*_*l*_ is the output current; *I*_*p*_ is the photocurrent, *I*_*diode*_ is the current flowing through diode.

As per Shockley equation, the diode current is expressed as:

2 $${I}_{diode}={I}_{d}\left[exp\left(\frac{q\left({V}_{l}+{I}_{l}{R}_{s}\right)}{a{k}_{B}T}\right)-1\right]$$

where, *a* is the ideality factor of diode; *T* is the cell temperature expressed in Kelvin; *k*_*B*_ is the Boltzmann constant with value of 1.3806 × 10^−23^ m^2^kg/s^2^K; *q* is the elementary charge with value of 1.602 × 10^−19^ C; ${V}_{l}$ signifies the voltage at output terminal and ${I}_{d}$ denotes the reverse saturation current of the diode.

The current going via shunt resistance can be expressed as follows, Eq. (3):

3 $${I}_{sh}=\frac{{V}_{l}+{{I}_{l}R}_{s}}{{R}_{sh}}$$

By combining Eqs. (1), (2) and (3), we arrive at:

4 $${I}_{l}={I}_{p}-{I}_{d}\left[exp\left(\frac{q\left({V}_{l}+{I}_{l}{R}_{s}\right)}{a{k}_{B}T}\right)-1\right]-\frac{{V}_{l}+{I}_{l}{R}_{s}}{{R}_{sh}}$$

It is very clear from Eq. (4) that five model parameters (${I}_{p}$, ${I}_{d}$, *a*,${R}_{s}$ and ${R}_{sh}$) must be estimated by using measured I–V data for the solar cell.

Similarly, the electrical behaviour of a PV module can be expressed by Eq. (5), as follows:

5 $${I}_{l}={I}_{p}{N}_{p}-{I}_{d}{N}_{p}\left[exp\left(\frac{q\left({V}_{l}+\frac{{R}_{s}{I}_{l}{N}_{s}}{{N}_{p}}\right)}{{a}_{1}{k}_{B}T{N}_{s}}\right)-1\right]-\frac{{V}_{l}+\frac{{R}_{s}{I}_{l}{N}_{s}}{{N}_{p}}}{\frac{{R}_{sh}{N}_{s}}{{N}_{p}}}$$

where* I*_*p*_ and *I*_*d*_ represent the photocurrent and saturation current of PV array, respectively; *N*_*s*_ depicts the number of solar cells connected in series; *N*_*p*_ depicts the number of solar cells connected in parallel. It is noteworthy that the more solar cells connected in parallel can increase the current of PV array, whereas more solar cells connected in series can provide greater output voltages.

### Objective function

As shown in the equivalent circuit of SDM, there are five unknown parameters represented in a solution vector of $X=({I}_{p}, {I}_{d}, a, {R}_{s}, {R}_{sh})$ to be identified. To solve the solar cell or PV module parameter estimation problem, an objective function needs to be defined and then optimized using a selected MA. Root mean square error (RMSE) is a popular objective function employed for the solar cell or PV module parameter estimation problem and it aims to minimize the errors between the experimental I-V data and simulated I-V data as follows, Eq. (6)^47^:

6 $$minimize\;(RMSE)=\sqrt{{\frac{1}{N}\sum_{n=1}^{N}{f}_{Solar cell/PV module}^{n}({V}_{l},{I}_{l},X)}^{2}}$$

For solar cell;

7 $$\left\{ {\begin{array}{*{20}c} {f_{Solar\, cell}^{n} \left( {V_{l} , I_{l} , X} \right) = I_{p} - I_{d} \left[ {exp\left( {\frac{{q\left( {V_{l} + I_{l} R_{s} } \right)}}{{ak_{B} T}}} \right) - 1} \right] - \frac{{V_{l} + I_{l} R_{s} }}{{R_{sh} }} - I_{l} } \\ {X = \left( {I_{p} , I_{d} , a, R_{s} , R_{sh} } \right) } \\ \end{array} } \right.$$

For PV module;

8 $$\left\{ {\begin{array}{*{20}c} {f_{PV\, module}^{n} \left( {V_{l} , I_{l} , X} \right) = I_{p} N_{p} - I_{d} N_{p} \left[ {exp\left( {\frac{{q\left( {V_{l} + \frac{{R_{s} I_{l} N_{s} }}{{N_{p} }}} \right)}}{{ak_{B} TN_{s} }}} \right) - 1} \right] - \frac{{V_{l} + \frac{{R_{s} I_{l} N_{s} }}{{N_{p} }}}}{{\frac{{R_{sh} N_{s} }}{{N_{p} }}}} - I_{l} } \\ {X = \left( {I_{p} , I_{d} , a, R_{s} , R_{sh} } \right) } \\ \end{array} } \right.$$

where *n* is the index of experimental point in given I-V data; *N* depicts the total numbers of observations in experimental I–V data; *X* is a decision variable vector consists of five unknown parameters to be optimized, where the search range of each parameter is defined in Table 2^47^.

**Table 2:** Search range of each parameter to be optimized.

Parameter (SI Unit)	For solar cell		For PV module	
	Lower bound	Upper bound	Lower bound	Upper bound
*I*_*p*_ (A)	0	1	0	10
*I*_*d*_ (µA)	0	0.5	0	50
*R*_*s*_ (Ω)	0.01	0.5	0.01	2
*R*_*sh*_ (Ω)	0.001	100	0.001	2000
*a*	1	2	1	100

## Estimation of solar cell/module

### Spotted hyena optimizer (SHO)

This algorithm is proposed by Dhiman and Kumar^38^ in the year of 2017. The inspiration for this algorithm is derived from the social behaviour of spotted hyena (Crocuta). Spotted hyenas are complex, smart, and incredibly social animals with a notorious behaviour. They have the capacity to combat indefinitely for territory and food. When a new food source is discovered, spotted hyenas generate a sound warning that is very similar to that of human laugh to interact with one another.

**Figure 3:**
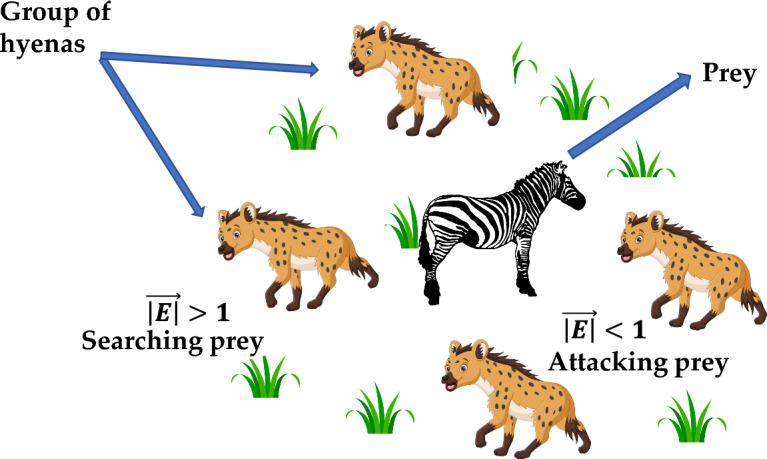
Searching and attacking behaviour of Spotted hyena.

**Figure 4:**
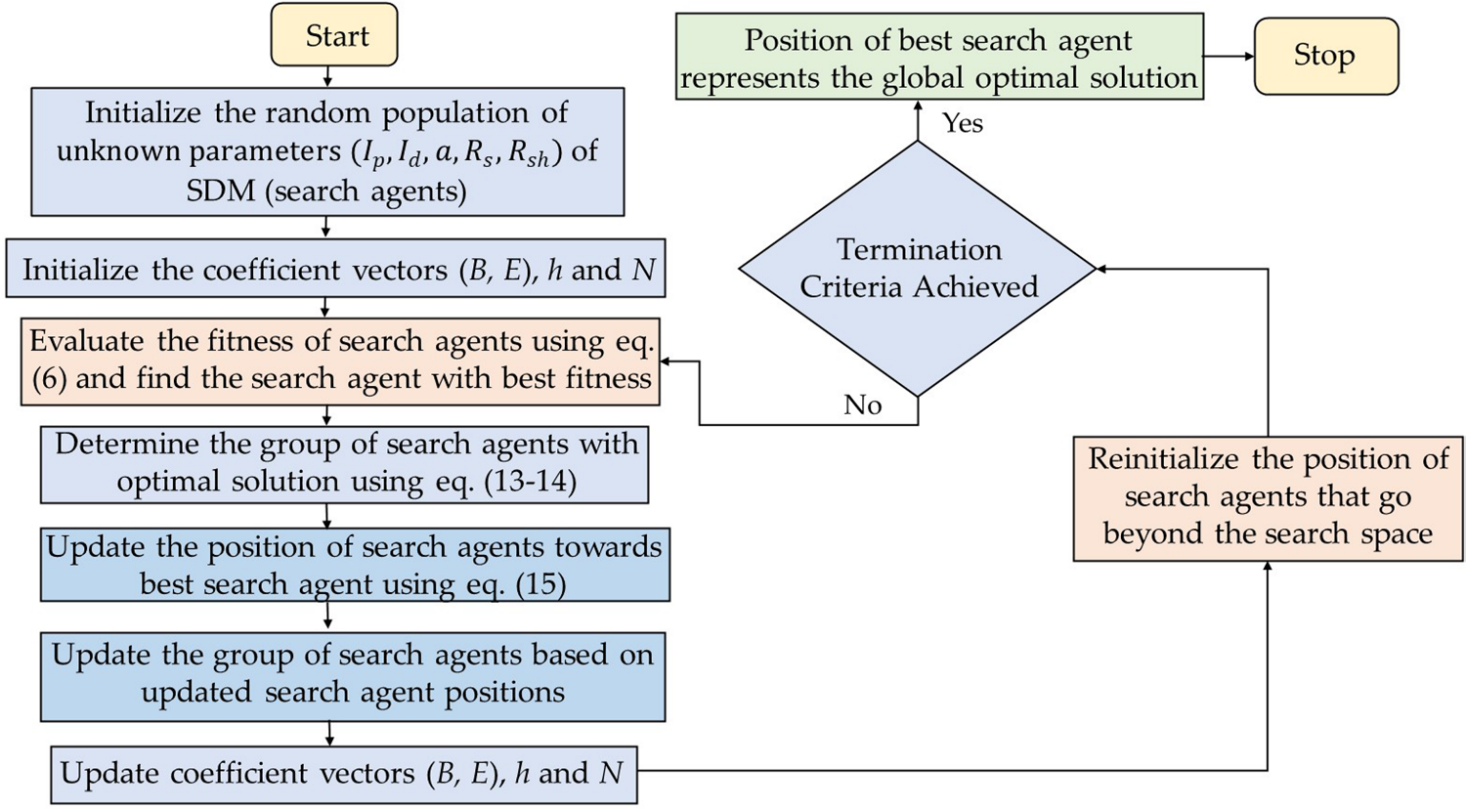
Process flow diagram of SHO algorithm.

Spotted hyenas adopt three types of mechanism: searching, encircling and attacking to acquire new food source. Figure 3 depicts the searching mechanism of SHO algorithm and Fig. 4 presents its process flow diagram.

#### Encircling prey

Spotted hyenas can recognize the position of prey with the help of sight or smell and surround it. The mathematical formulation of this mechanism is defined by the following Eqs. (9) and (10).

9 $$\overrightarrow{{D}_{h}}= \left|\overrightarrow{B}.\overrightarrow{{P}_{p}}\left(x\right)- \overrightarrow{P}\left(x\right)\right|$$

10 $$\overrightarrow{P}\left(x+1\right)= \overrightarrow{{P}_{p}} \left(x\right)- \overrightarrow{E}. \overrightarrow{{D}_{h}}$$

where, $\overrightarrow{{D}_{h}}$ represents the distance between spotted hyena and prey, the current iteration is denoted by $x$. $\overrightarrow{B}$ and $\overrightarrow{E}$ represents coefficient vector. Position of prey is represented by $\overrightarrow{{P}_{p}}$ , while $\overrightarrow{P}$ denotes the position vector of spotted hyena.

#### Hunting

Spotted hyenas generally live and hunt in teams, relying on a network of trusted groups and their ability to identify prey location. The hunting mechanism can be defined mathematically as follows:

11 $$\overrightarrow{{D}_{h}}= \left|\overrightarrow{B}.\overrightarrow{{P}_{h}}- \overrightarrow{{P}_{k}}\right|$$

12 $$\overrightarrow{{P}_{k}}= \overrightarrow{{P}_{h}} - \overrightarrow{E}. \overrightarrow{{D}_{h}}$$

13 $$\overrightarrow{{C}_{h}}= \overrightarrow{{P}_{k}}+\overrightarrow{{P}_{k+1}}+\dots + \overrightarrow{{P}_{k+N}}$$

where, $\overrightarrow{{P}_{h}}$ denotes the location of first best spotted hyena. The location of another spotted hyena is presented by $\overrightarrow{{P}_{k}}$ . N represents the number of spotted hyenas and is evaluated as:

14 $$N=number\;of\;solutions\,[\overrightarrow{{P}_{h}}, {\overrightarrow{P}}_{h+1},{\overrightarrow{P}}_{h+2},\dots \dots \dots \dots ,({\overrightarrow{P}}_{h}+\overrightarrow{M})]$$

where $\overrightarrow{M}$ is the random vector defined in the range [0.5, 1].

#### Attacking prey

The attacking mechanism of spotted hyena can be presented mathematically as

15 $$\overrightarrow{P}\left(x+1\right)= \frac{\overrightarrow{{C}_{h}}}{N}$$

where the function of $\overrightarrow{P}\left(x+1\right)$ is to save the best solution ever found and $\overrightarrow{{C}_{h}}$ denotes the cluster of number of best optimized solution.

### Sooty tern optimization (STO)

**Figure 5:**
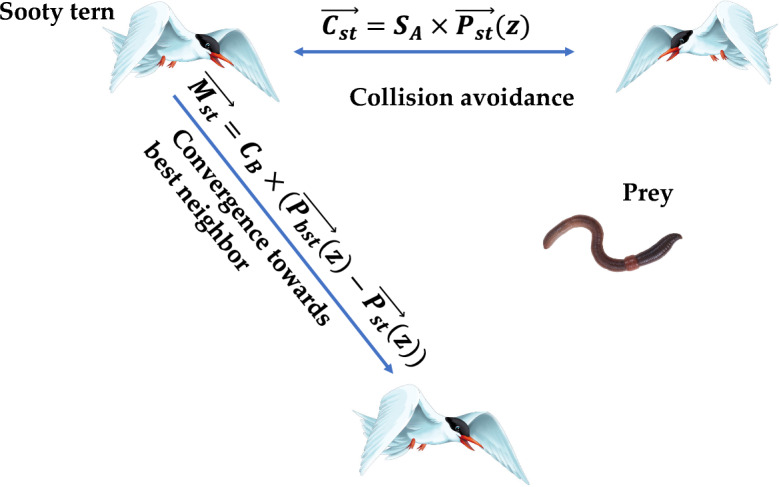
Searching behavior of sooty tern optimization algorithm.

**Figure 6:**
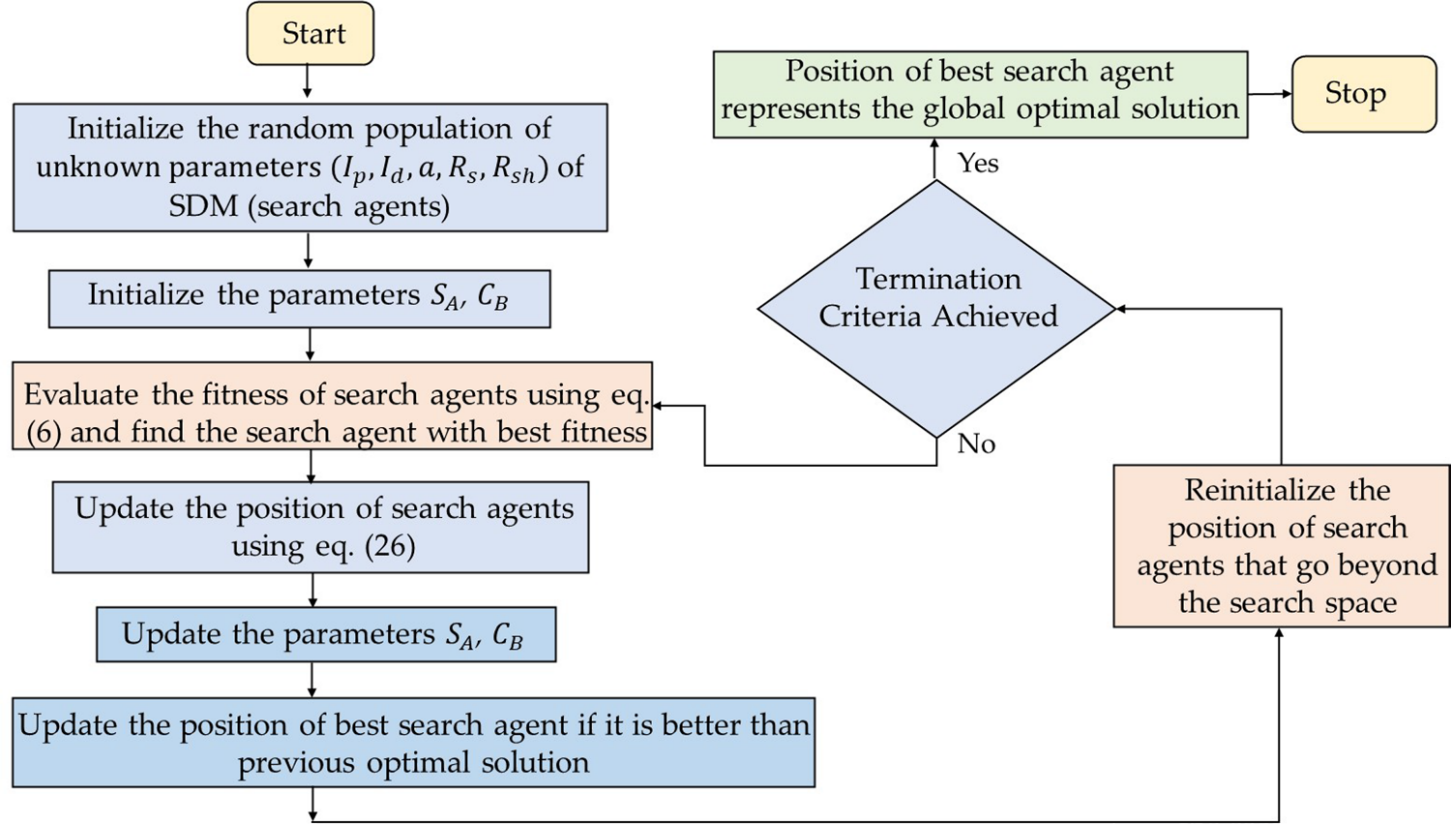
Process flow diagram of STO algorithm.

Sooty tern optimization (STO) algorithm is a bio-inspired algorithm proposed by Dhiman and Kaur^39^, in 2019. The algorithm is inspired by the migration and the attacking behavior of sooty tern in our mother nature. Figure 4 illustrates the searching behavior of STO algorithm. Sooty tern are intelligent sea birds belongs to the Laridae family and are mostly found in tropical oceans across the world. These birds are omnivorous and found in different sizes and masses. Sooty tern belongs to the oviparous family and mostly lives in sea and comes only for breeding on the island. These birds migrate in groups from one place to another place so as to search the adequate food sources for their survival. In a group, all birds follow the best sooty bird in order to reach their food sources in optimum time. Although, these birds have unique migration movement they also have unique attacking mechanism. These birds use spiral movement during their attack in the air. Figures 5 and 6 shows the searching behavior and process flow of STO algorithm. The mathematical formulation of STOA search mechanism is provided as follows:

*Step 1* Initialize the position of sooty tern (search agents) arbitrarily in the defined space:

16 $${\overrightarrow{P}}_{s}=\left({\overrightarrow{p}}_{1}, {\overrightarrow{p}}_{2},\dots \dots \dots \dots \dots \dots \dots {\overrightarrow{p}}_{n}\right)$$

where, n signifies the space dimension.

*Step 2* Evaluate the cost of all sooty tern, depending on the problem (minimization or maximization) the position of best sooty tern (${\overrightarrow{p}}_{bs}$) represents the best search agent.

*Step 3* Initialize the parameters S_A_ and C_B_ which are responsible for the movement of sooty tern in the search space. These parameters are defined as:

17 $$S_{A} = C_{f} - \left( {z*\left( {{\raise0.7ex\hbox{${C_{f} }$} \!\mathord{\left/ {\vphantom {{C_{f} } {Max_{{iterations}} }}}\right.\kern-\nulldelimiterspace} \!\lower0.7ex\hbox{${Max_{{iterations}} }$}}} \right)} \right)$$

where, ${C}_{f}$ is the controlling variable whose value is linearly decreased from ${C}_{f}$ to zero,

$${\text{z }} = \, 0,{ 1},{ 2},{ 3}, \, \ldots \ldots \ldots \ldots \ldots \ldots \ldots \ldots \ldots .,Max_{iterations} .$$

18 $${C}_{B}=0.5*{R}_{and}$$

where, ${R}_{and}$ is the arbitrary number in the range [0,1].

*Step 4* Update the position of sooty tern based on the following equations:

19 $${x}^{^{\prime}}={R}_{adious}*\mathrm{sin}\left(i\right)$$

20 $${y}^{^{\prime}}={R}_{adious}*\mathrm{cos}\left(i\right)$$

21 $${z}^{^{\prime}}={R}_{adious}*i$$

22 $$r=u*{e}^{kv}$$

where, ${R}_{adious}$ represents the radius of the spiral movement, *i* is the variable in the range [$0\le k\le 2\pi$], *u* and *v* are the constant parameters.

23 $${\overrightarrow{C}}_{s}={S}_{A}*{p}_{S}$$

24 $${\overrightarrow{M}}_{s}={C}_{B}*\left({\overrightarrow{p}}_{bs}-{\overrightarrow{p}}_{s}\right)$$

25 $${\overrightarrow{D}}_{s}={\overrightarrow{C}}_{S}+{\overrightarrow{M}}_{s}$$

26 $${\overrightarrow{p}}_{s}=\left({\overrightarrow{D}}_{s}*\left({x}^{^{\prime}}+{y}^{^{\prime}}+{z}^{^{\prime}}\right)\right)*{\overrightarrow{p}}_{bs}$$

*Step 5* Update the parameters ${S}_{A}$ and ${C}_{B}$.

*Step 6* Update the position of best sooty tern if it is better than the previous optimal solution.

*Step 7* Reinitialize the position of sooty tern that go beyond the defined space.

*Step 8* The algorithm terminates when the minimal error or maximum number of iterations is reached. Alternatively, resume steps (3) to (7).

*Step 9* The location of finest sooty tern (${\overrightarrow{p}}_{bs}$) reflects the global ideal solution.

### Aquila optimization (AO)

**Figure 7:**
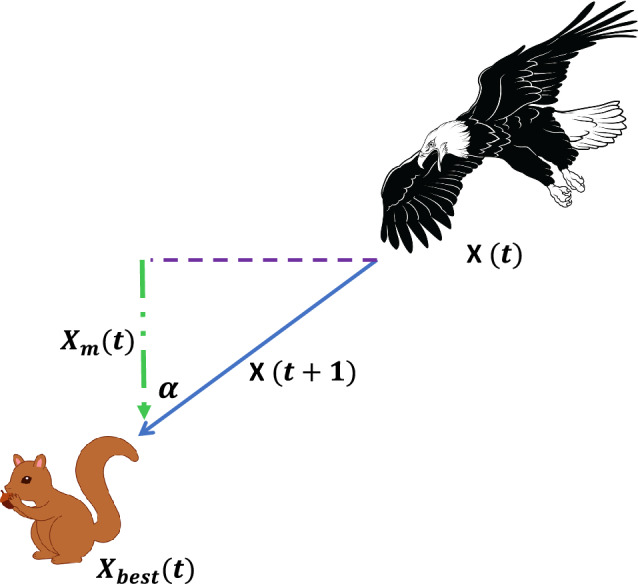
Searching behaviour of aquila optimization algorithm.

**Figure 8:**
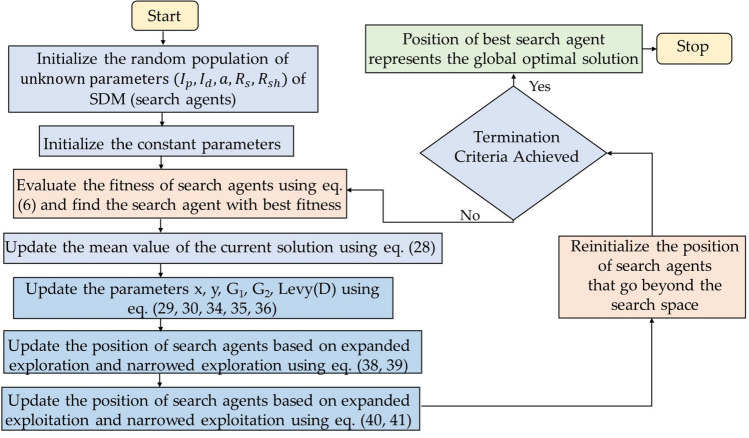
Process flow diagram of AO algorithm.

Aquila optimization algorithm (AO) is a nature inspired population-based algorithm proposed by Abualigah et al*.*^40^ in 2021. Aquila, also commonly known as eagles, are dark colored birds that belong to the group Accipitridae and are known for their sharp and intelligent hunting behavior. These wild birds are fast, agile and has large sturdy feet with sharpened talons, which help them to attack and grab their prey over longer distances. The main source of food of these birds are squirrels, rabbits, hares, marmots, deeps and other small ground animals. The algorithm is inspired from the skillful hunting behavior of aquila that can be considered as the second-best hunting behavior after human beings. AO is mathematically modeled around four hunting methods of aquila which includes high soar with vertical stoop, short glide attack with contour flight, slow descent attack through low flight, grab prey through walk. These four attacking approaches are mathematically modeled and designed to showcase each step of hunt through expanded exploration, narrowed exploration, expanded exploitation and narrowed exploitation in order to maintain equilibrium between exploration and exploitation. The searching behavior and process flow of AO algorithm is revealed in Figs. 7 and 8. The mathematical representation of AO is given as follows:

*Step 1* Initialize the population of aquila (search agent) arbitrarily in the defined space:

27 $$\overrightarrow{X}=\left({\overrightarrow{x}}_{1}, {\overrightarrow{x}}_{2},\dots \dots \dots \dots \dots \dots \dots {\overrightarrow{x}}_{n}\right)$$

where, n signifies the space dimension.

*Step 2* Evaluate the cost of all aquila, depending on the problem (minimization or maximization) the position of best aquila (${X}_{best}$) represents the finest search agent.

*Step 3* Initialize the constant parameters of AO.

*Step 4* Update the mean value of the current solution ${X}_{M}$.

28 $${X}_{M}\left(t\right)=\frac{1}{M}\sum_{i=1}^{M}{X}_{i}\left(t\right), \forall i=\mathrm{1,2},\dots \dots \dots \dots ., n$$

where, M is the number of solution.

*Step 5* Update the parameters *x*, *y*, G_1_, G_2_, Levy(D) which are defined as:

29 $$x=r*\mathrm{sin}\left(\theta \right)$$

30 $$y=r*\mathrm{cos}\left(\theta \right)$$

31 $$\mathrm{where}, r={r}_{1}+U*{D}_{1}$$

32 $$\theta =-\omega *{D}_{1}+{\theta }_{1}$$

33 $${\theta }_{1}=\frac{3*\pi }{2}$$

r_1_ is defined in the range 1 to 20, U is 0.00565, D_1_ lies between 1 to length of the search space, $\omega$ is 0.005.

34 $${G}_{1}=2*rand-1$$

35 $${G}_{2}=2*\left(1-\frac{t}{T}\right)$$

where, *rand* is the arbitrary number in the range [0, 1], t and T is the existing iteration and the maximum number of iterations.

36 $$Levy\left(D\right)=s*\frac{u*\sigma }{{\left|v\right|}^{\frac{1}{\beta }}}$$

where, s is the constant value 0.01, *u* and *v* are random values lies in the range [0,1], $\beta$ is constant value 1.5 and $\sigma$ is defined as:

37 $$\sigma = \left( {\frac{{\Gamma \left( {1 + \beta } \right)*sine\left( {\frac{\pi \beta }{2}} \right)}}{{\Gamma \left( {\frac{1 + \beta }{2}} \right)*\beta *2^{{\left( {\frac{\beta - 1}{2}} \right)}} }}} \right)$$

*Step 6* Update the position of aquila as per the following equations:if $t\le \left(\frac{2}{3}\right)*T$if rand $\le 0.5$

Update the position of aquila using expanded exploration $\left({X}_{1}\right)$:

38 $${X}_{1}\left(t+1\right)={X}_{best}\left(t\right)*\left(1-\frac{t}{T}\right)+\left({X}_{M}\left(t\right)-{X}_{best}\left(t\right)*rand\right)$$

where, ${X}_{1}\left(t+1\right)$ is the solution of the next iteration of *t*.else,

**Table:** 

if	cost (X_1_(t + 1)) < cost (X(t)) X(t) = X_1_(t + 1)
if	cost (X_1_(t + 1) < cost(X_best_(t)) X_best_(t) = X_1_(t + 1)

Update the position of aquila using narrowed exploration $\left({X}_{2}\right)$:

39 $${X}_{2}\left(t+1\right)={X}_{best}\left(t\right)*Levy\left(D\right)+{X}_{R}\left(t\right)+\left(y-x\right)*rand$$

where, ${X}_{2}\left(t+1\right)$ is the solution of the next iteration of *t*.else:if $rand\le 0.5$

**Table:** 

if	cost (X_2_(t + 1)) < cost (X(t)) X(t) = X_2_(t + 1)
if	cost (X_2_(t + 1) < cost(X_best_(t)) X_best_(t) = X_2_(t + 1)

Update the position of aquila using expanded exploitation $\left({X}_{3}\right)$:

40 $${X}_{3}\left(t+1\right)=\left({X}_{best}\left(t\right)-{X}_{M}(t)\right)*\alpha -rand+\left(\left(UB-LB\right)*rand+LB\right)*\delta$$

where, ${X}_{3}\left(t+1\right)$ is the solution of the next iteration of *t*.else:

**Table:** 

if	cost (X_3_(t + 1)) < cost (X(t)) X(t) = X_3_(t + 1)
if	cost (X_3_(t + 1) < cost(X_best_(t)) X_best_(t) = X_3_(t + 1)

Update the position of aquila using narrowed exploitation $\left({X}_{4}\right)$:

41 $${X}_{4}\left(t+1\right)=QF*{X}_{best}\left(t\right)-\left({G}_{1}*X\left(t\right)*rand\right)-{G}_{2}*Levy\left(D\right)+rand*{G}_{1}$$

where,

42 $$QF\left(t\right)={t}^{\frac{2*rand-1}{{\left(1-T\right)}^{2}}}$$

where, ${X}_{4}\left(t+1\right)$ is the solution of the next iteration of *t*.

**Table:** 

if	cost (X_4_(t + 1)) < cost (X(t)) X(t) = X_4_(t + 1)
if	cost (X_4_(t + 1) < cost(X_best_(t)) X_best_(t) = X_4_(t + 1)

*Step 7* Reinitialize the position of aquila that go beyond the defined space.

*Step 8* The algorithm terminates when the minimal error or maximum number of iterations is reached. Alternatively, resume steps (4)–(6).

*Step 9* The location of finest aquila (${X}_{best}$) signifies the global optimal solution.

### Harris hawks optimization (HHO)

**Figure 9:**
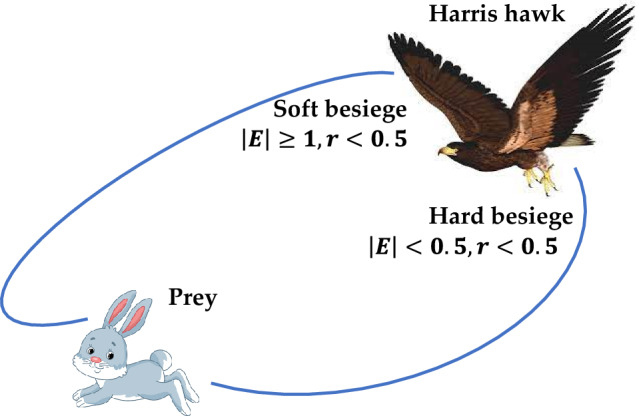
Searching behaviour of Harris Hawk optimizer.

**Figure 10:**
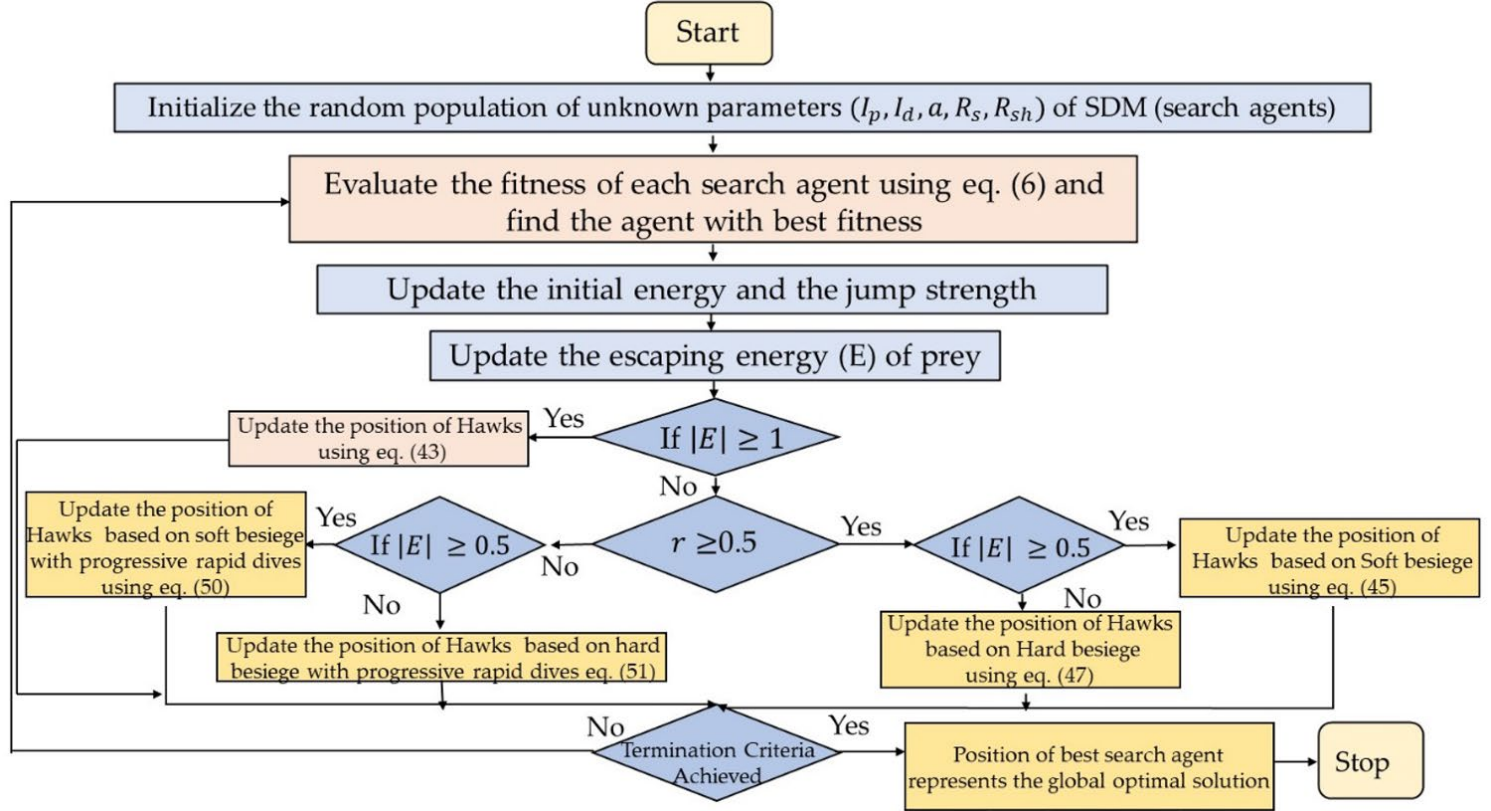
Process flow diagram of HHO algorithm.

Harris hawks optimization algorithm is inspired from the collaborative behaviour and chasing style of Harris hawk^41^. Harris hawks can exhibit a wide range of chasing styles based on the dynamic nature of situations and the prey's escaping styles. Harris hawks finds the optimal solution by using two phases: exploitation and exploration. Figures 9 and 10 depicts HHO's searching behaviour and its process flow diagram.

#### Exploration phase

In HHO each Harris’ hawks represents the candidate solution, and the best candidate solution in each phase is regarded to be the intended near the prey. In HHO, Harris’ hawks perch at random in various locations and wait for prey using one of two approaches. If we assume an equal chance q for each perching strategy, they will perch relying on the locations of other family members (to be close enough to them when attacking) and the rabbit, as shown in Eq. (43).

43 $$X\left( {t + 1} \right) = \left\{ {\begin{array}{*{20}l} {X_{rand} \left( t \right) - r_{1} \left| {X_{rand} \left( t \right) - 2r_{2} X\left( t \right)} \right|} \hfill & {q \ge 0.5} \hfill \\ {(X_{rabbit} \left( t \right) - X_{m} \left( t \right)) - r_{3} \left( {LB + r_{4} \left( {UB - LB} \right)} \right) } \hfill & {q < 0.5} \hfill \\ \end{array} } \right.$$

where, $X(t)$ signifies the current position of hawks, ${X}_{rand}\left(t\right)$ is the randomly selected hawk at ${t}^{th}$ iteration, ${r}_{1}, {r}_{2}, {r}_{3},$ and ${r}_{4}$ represents the random number in the range of [0,1]. ${X}_{rabbit}(t)$ is the location of rabbit. $UB$ and $LB$ defines the upper and lower bound of the variables. ${X}_{m}$ denotes the average location of hawks.

The average location of hawks can be computed by using Eq. (44).

44 $${X}_{m}\left(t\right)=\frac{1}{N}\sum_{i=1}^{N}{X}_{i}(t)$$

where, $N$ signifies the total number of hawks.

#### Exploitation phase

The Harris' hawks perform the surprise pounce in this phase by targeting the intended prey identified in the previous stage. Prey, on the other hand, frequently attempts to flee dangerous situations. As a result, distinct chasing patterns emerge in real-world situations. The HHO proposes four strategic options to model the attacking stage based on prey escaping behaviours and chasing strategies of Harris' hawks. These are soft besiege, hard besiege, soft besiege with progressive rapid dives and hard besiege with progressive rapid dives.

In the first strategy of soft besiege prey didn’t escape from the hawks because their energy has been drained and it happens when escaping energy (E) and the chance of escape (r) both are greater than equal to 0.5. The mathematical model of this strategy is described as

45 $$X\left( {t + 1} \right) = \Delta X\left( t \right) - E\left| {JX_{rabbit} \left( t \right) - X\left( t \right)} \right|$$

46 $$\Delta X\left(t\right)={X}_{rabbit}-X(t)$$

where $\Delta X$ presents the difference between current location and position vector of rabbit in *t*th iteration while $J=2(1-{r}_{5})$ and ${r}_{5}$ is the random number defined in the range [0, 1].

In the second strategy of hard besiege where $\left|E\right|<0.5$ and $r\ge 0.5$, the prey has exhausted, therefore, it can’t escape from the hawk. The position of hawks are defined as:

47 $$X(t+1)={X}_{rabbit}(t)-E*\left|\Delta X(t)\right|$$

In the third strategy of soft besiege with progressive rapid dives where $\left|E\right|\ge 0.5$ and $r<0.5$ prey has energy to escape from the hawks and hawks follow the soft besiege. This strategy is mathematically defined as:

48 $$M={X}_{rabbit}(t)-E\left|J*{X}_{rabbit}(t)-X(t)\right|$$

49 $$N=M+S*LF(D)$$

where S represents random vector, D signifies the problem diminution and LF is the levy flight function.

The hawks’ updated position can be modelled as:

50 $$X(t+1)=\left\{\begin{array}{c}M\, if\, F(M)<F(X(t))\\ N\,if\,F(N)<F(X(t))\end{array}\right.$$

In the fourth strategy of hard besiege with progressive rapid dives prey can’t escape because of less energy and hawks follow hard besiege where $\left|E\right|<0.5$ and $r<0.5$. The mathematical model of this strategy is defined as:

51 $$X(t+1)=\left\{\begin{array}{c}M\, if\, F({M}^{\mathrm{^{\prime}}})<F(X(t))\\ {N}^{\mathrm{^{\prime}}}\,if\,F({N}^{\mathrm{^{\prime}}})<F(X(t))\end{array}\right.$$

52 $${\text{where}},\;\;\;M^{\prime} = X_{rabbit} \left( t \right) - E\left| {J{*}X_{rabbit} \left( t \right) - X_{m} \left( t \right)} \right|$$

53 $${N}^{\mathrm{^{\prime}}}={M}^{\mathrm{^{\prime}}}+S*LF(D)$$

### Wild horse optimization (WHO)

**Figure 11:**
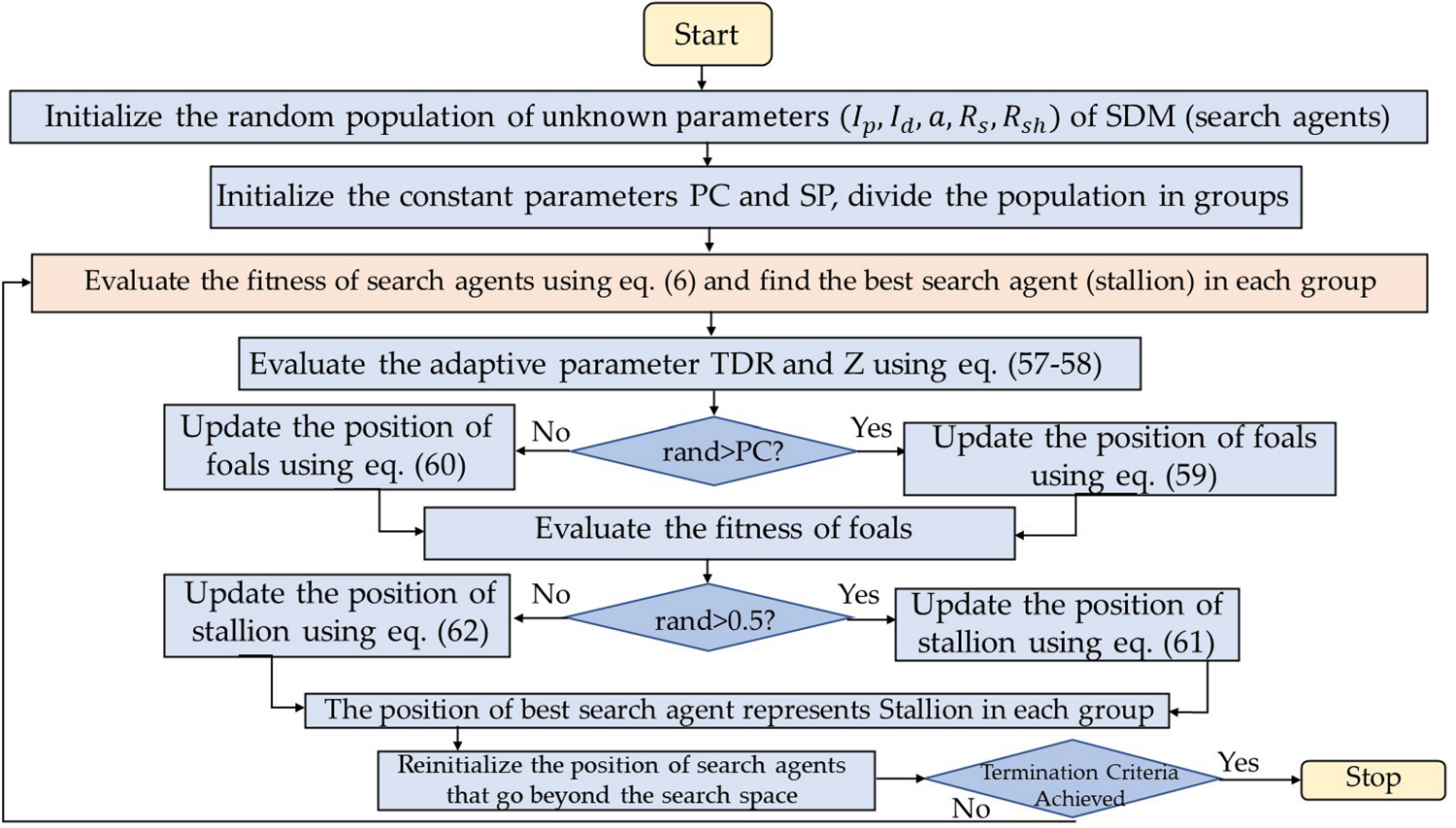
Process flow diagram of WHO algorithm.

Wild horse optimization (WHO) algorithm is a nature inspired algorithm proposed by Naruei and Keynia^42^ in 2021. The algorithm gets its motivation from the behavior of the wild horses. It is a population based gradient free stochastic algorithm that considers the problem as black box and finds near optimal solution for wide range of complex optimization problems. Horses are mostly classified around their social behavior as territorial and non-territorial horses. WHOA focuses on non-territorial horses where the horses live in family or social groups which includes a stallion and several other mares and foals. The algorithm mathematically models the grazing, mating, dominance and leadership quality of wild horses to solve optimization problems. Foal horses have more grazing in the initial stages of their life and less as they get older. Foals have an interesting behavior that they leave their parent group before puberty in order to prevent being mated from their father. Stallion is the most dominant horse in the group and all other mares and foals follow or change their direction of movement with respect to stallion. Figure 11 presents the process flow diagram of WHO algorithm. The mathematical representation of the WHOA is provided as follows:

*Step 1* Initialize the population of horses (search agents) arbitrarily in the definite space:

54 $$\overrightarrow{X}=\left({\overrightarrow{x}}_{1}, {\overrightarrow{x}}_{2},\dots \dots \dots \dots \dots \dots \dots {\overrightarrow{x}}_{n}\right)$$

where, n signifies the space dimension.

*Step 2* Initialize the constant parameters crossover percentage (PC) and stallion percentage (PS) and divide the population into several groups where the number of groups are

55 $$G=\left[N\times PS\right]$$

where, G is the number of stallions and N is the population size. The number of members in each group are

56 $$GM=\left[N-G\right]$$

*Step 3* Evaluate the cost of all the horses, depending on the problem (minimization or maximization), the position of the best horses represents stallions in each group.

*Step 4* Evaluate the adaptive parameter *TDR* as follows:

57 $$TDR=1-iter\times \left(\frac{1}{maxiter}\right)$$

*Step 5* Find out another adaptive parameter *Z* as:

58 $$Z={R}_{2}\Theta IDX+{\overrightarrow{R}}_{3}\Theta \left(\sim IDX\right)$$

where,

$$P={\overrightarrow{R}}_{1}<TDR;IDX=\left(P==0\right)$$

where, ${R}_{2}$ is a arbitrary number with unchanging distribution in range [0,1],

${\overrightarrow{R}}_{1}$ & ${\overrightarrow{R}}_{3}$ are random vectors with uniform distribution [0, 1],

*P* is a vector that contains 0 and 1 which equals the dimension of the search problem.

*Step 6* Update the position of foals and stallion of each group as per the following equations:for number of foals of any group

$$if\, rand>PC$$

59 $${X}_{i,G}^{j}=2Zcos\left(2\pi RZ\right)*\left({Stallion}^{j}-{X}_{i,G}^{j}\right)+{Stallion}^{j}$$

where, ${X}_{i,G}^{j}$ is the position of foal, ${Stallion}^{j}$ is the position of Stallion, R is the uniform random number in the range [-2, 2].else

60 $${X}_{G,k}^{p}=Crossover\left({X}_{G,i}^{q},{X}_{G,j}^{z}\right)\,\,\,\,\,\,\,i\ne j\ne k,p=q=end$$

end for each stallion of each group

$$if\, rand>0.5$$

61 $${\overline{Stallion} }_{{G}_{i}}=2Zcos\left(2\pi RZ\right)\times \left(WH-{Stallion}_{{G}_{i}}\right)+WH$$

else

62 $${\overline{Stallion} }_{{G}_{i}}=2Zcos\left(2\pi RZ\right)\times \left(WH-{Stallion}_{{G}_{i}}\right)-WH$$

where, WH is the position of the water hole, ${\overline{Stallion} }_{{G}_{i}}$ is the position of the leader of the *i*^th^ group.

*Step 7* If the position of Stallion if better than its previous position then update the position of stallion, if the position of foal in any group is better than stallion position then exchange foal and stallion position as per the following equation:

63 $$Stallion_{{G_{i} }} = \left\{ {\begin{array}{*{20}l} {X_{G,i} } \hfill & {if\;cost\left( {X_{G,i} } \right) < cost\;\left( {Stallion_{{G_{i} }} } \right)} \hfill \\ {Stallion_{{G_{i } }} } \hfill & {if\;cost\left( {X_{G,i} } \right) > cost\;\left( {Stallion_{{G_{i} }} } \right)} \hfill \\ \end{array} } \right.$$

*Step 8* Reinitialize the position of horses that go beyond the defined space.

*Step 9* The algorithm terminates when the minimal error or maximum number of iterations is reached. Alternatively, resume steps (4) to (8).

*Step 10* The location of finest Stallion signifies the global optimal solution.

### Arithmetic optimization algorithm (AOA)

**Figure 12:**
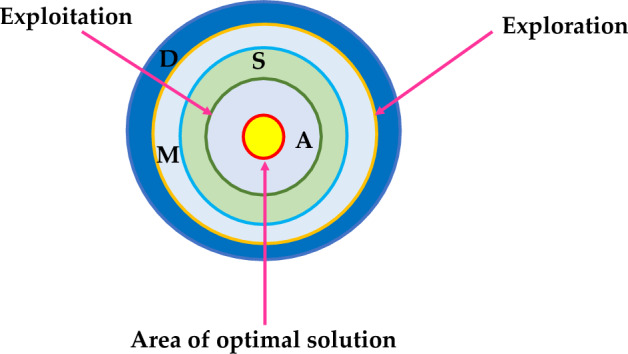
Searching mechanism of Arithmetic Optimization.

**Figure 13:**
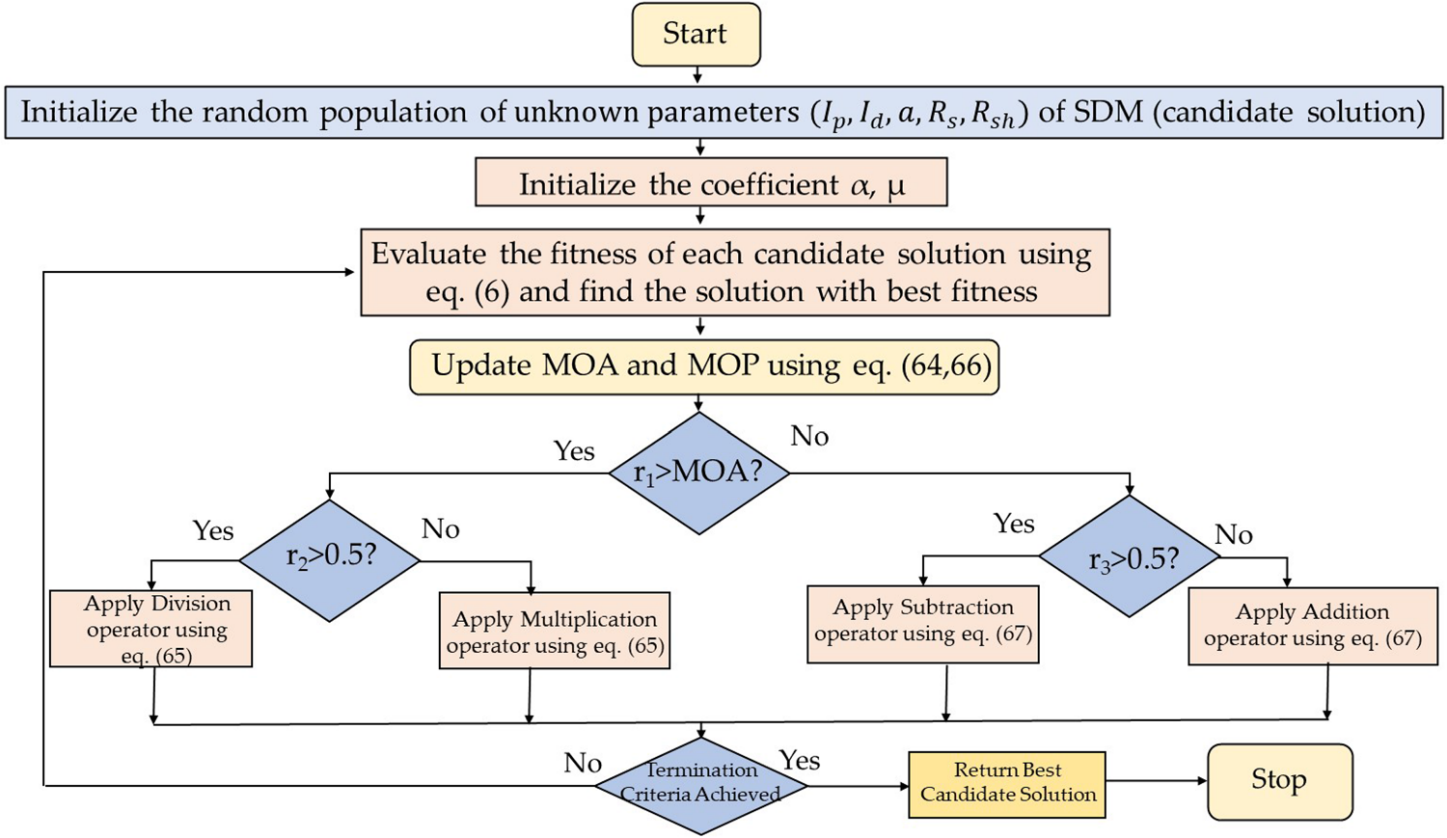
Process flow diagram of AOA algorithm.

Authors in^43^ anticipated a novel optimization algorithm known as arithmetic optimization algorithm (AOA). AOA makes use of the distribution behaviour of the major arithmetic operators in mathematics that includes multiplication (*M*), division (*D*), subtraction (*S*), and addition (*A*). This optimization mainly consists of two stages: exploration and exploitation. Exploration pertains to the use of search agents of an algorithm to cover a large portion of the search space to prevent local solutions. Exploitation enhances the precision of the found solutions throughout the exploration stage. Figures 12 and 13 depicts the AOA searching technique and its process flow diagram. The math optimizer accelerated (MOA) function maintains balance between exploration and exploitation and is defined as:

64 $$MOA(Iter)=Min+Iter*\left(\frac{Max-Min }{Max\_Iter}\right)$$

where Iter and Max_Iter signifies the current iteration and the maximum number of iterations, Min and Max represents the accelerated function minimum and maximum values.

#### Exploration stage

In AOA, exploration operators randomly explore the search area on many regions and tend to produce a best optimal solution focused on two major search strategies (Division (*D*) search strategy and Multiplication (*M*) search strategy), as modelled in Eq. (64).

65 $$x_{i,j} \left( {C \_Iter + 1} \right) = \left\{ {\begin{array}{*{20}l} {best\left( {x_{j} } \right) \div \left( {MOP + \epsilon } \right) \times \left( {\left( {UB_{j} - LB_{j} } \right) \times \mu + LB_{j} } \right), } \hfill & {r2 < 0.5} \hfill \\ {best\left( {x_{j} } \right) \times MOP \times \left( {\left( {UB_{j} - LB_{j} } \right) \times \mu + LB_{j} } \right),} \hfill & {otherwise} \hfill \\ \end{array} } \right.$$

where $U{B}_{j}$ and $L{B}_{j}$ signifies the upper and lower bound of *j*th location. ${x}_{i,j}\left(C \_Iter+1\right)$ denotes the *i*th solution in next iteration at *j*th location. $\mu$ indicates the control parameter used for adjusting the search strategy. $\epsilon$ denotes a small integer number. $best\left({x}_{j}\right)$ represents the *j*th location of the best optimal solution found so far. $MOP$ is the math optimizer probability and is defined as:

66 $$MOP(Iter) = 1 - \frac{{Iter^{{1/\alpha }} }}{{Max\_Iter^{{1/\alpha }} }}$$

#### Exploitation stage

In this stage two mathematical operators’ subtraction (*S*) and addition (*A*) are used as they produce highly dense result. These operators are capable to reach the target due to their low dispersion characteristic. The exploitation strategy can be represented mathematically by using Eq. (65).

67 $$x_{i,j} \left( {C \_Iter + 1} \right) = \left\{ {\begin{array}{*{20}l} {best\left( {x_{j} } \right) - MOP \times \left( {\left( {UB_{j} - LB_{j} } \right) \times \mu + LB_{j} } \right), } \hfill & {r3 < 0.5} \hfill \\ {best\left( {x_{j} } \right) \times MOP \times \left( {\left( {UB_{j} - LB_{j} } \right) \times \mu + LB_{j} } \right), } \hfill & {otherwise} \hfill \\ \end{array} } \right.$$

This phase makes the most of the search space by conducting a thorough search. In this stage (first rule in Eq. (65)), the first operator (S) is conditioned by $r3<$ 0.5, and the other operator (A) is ignored until this operator completes its current task.

### Atom search optimization (ASO) algorithm

**Figure 14:**
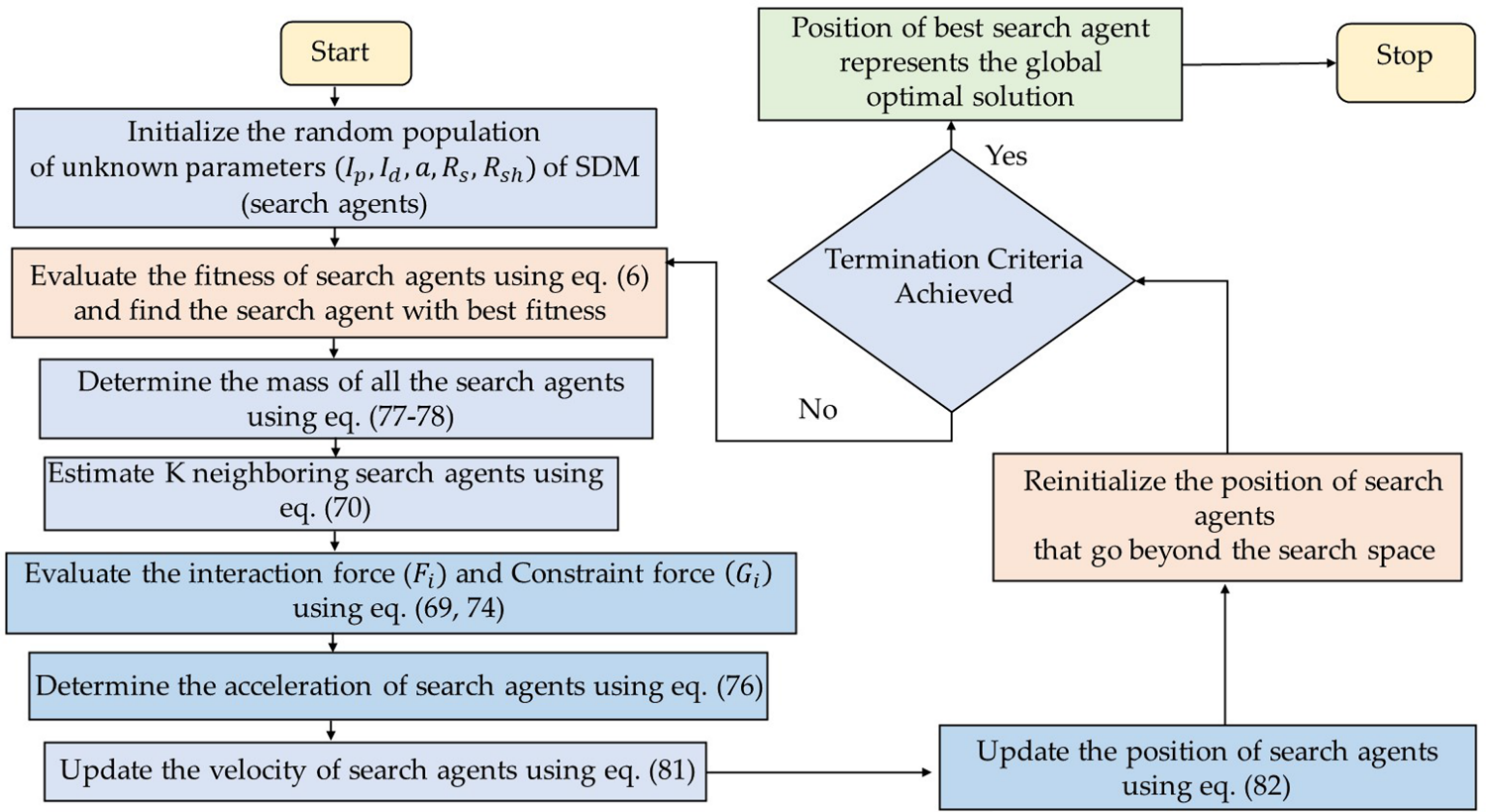
Process flow diagram of ASO algorithm.

Atom search optimization (ASO) is a physics-based metaheuristic optimization technique that mimics the theory of molecular dynamics^44^. In ASO, each atom's location within the search space symbolizes a solution as analysed by its mass, with a finest solution implying a heavier mass and vice versa. All atoms in the population will attract or repel one another based on their distance from one another, causing the lighter atoms to flock toward the heavier ones. Heavier atoms have less speed, which enables them to rigorously demand a new local solution. While lighter atoms accelerate more rapidly due to low mass, they search extensively for new promising regions throughout the search space. Figure 14 shows the process flow diagram of ASO algorithm.

#### Mathematical representation of interaction force

The priming power of atomic motion is the interaction force resulting from the L-J potential. At *t*th iteration, the interaction force generated by *j*th atom on *i*th can be represented by using Eq. (68) as follows:

68 $$F_{ij} \left( t \right) = \frac{24\varepsilon \left( t \right)}{{\sigma \left( t \right)}}2\left( {\frac{\sigma \left( t \right)}{{r_{ij} \left( t \right)}}} \right)^{13} - \left( {\frac{\sigma \left( t \right)}{{r_{ij} \left( t \right)}}} \right)^{7} \frac{{r_{ij} \left( t \right)}}{{r_{ij}^{d} \left( t \right)}}$$

where, $\sigma$ signifies the collision diameter, $\varepsilon$ represents the magnitude of the interactive force. ${r}_{ij}(t)$ is the distance between *j*th and *i*th atom at time $t$. ${F}_{ij}$ denotes the interactive force.

69 $${F}_{i}^{d}\left(t\right)=\sum_{j\varepsilon Kbest}{rand}_{j}{F}_{ij}^{d}\left(t\right)$$

where, $kbest$ signifies the atoms with best fitness values and *K* maintains the balance between exploration and exploitation and decreases gradually over the course of iterations and is defined as:

70 $$K\left( t \right) = N - \left\{ {\left( {N - 2} \right){*}\sqrt{\frac{t}{T}} { }} \right\}$$

where, N is the total number of atoms.

However, in order to solve the optimization problem Eq. (71) is revised as:

71 $${F}_{ij}^{^{\prime}}\left(t\right)=-n(t)[2({h}_{ij}\left(t\right){)}^{13}-({h}_{ij}\left(t\right){)}^{7}]$$

where, $n(t)$ denotes the depth function implemented for repositioning the repulsion or attraction region, which can be signified as follows:

72 $$n\left(t\right)=\alpha (1-\frac{t-1}{T}{)}^{3}{e}^{\frac{-20t}{T}}$$

where, $\alpha$ represents depth weight and *T* denotes the number of iterations.

#### Mathematical representation of geometric constraint

In molecular dynamics, the geometric constraint is very crucial in atomic motion. Assume that each atom in ASO has a covalence bond with the finest atom for the sake of simplicity. The constraint of ${i}^{th}$ atom can be written as follows:

73 $${\theta }_{i}\left(t\right)=\left[{\left|{X}_{i}\left(t\right)-{X}_{best}\left(t\right)\right|}^{2}-{b}_{i,best}^{2}\right]$$

where, ${X}_{i}\left(t\right)$ is the location of *i*th atom at time *t*, ${b}_{i,best}$ denotes the fixed bond length in between ${i}^{th}$ atom and best atom, ${X}_{best}$ represents the location of best atom found so far. The constraint force is defined as:

74 $${G}_{i}^{d}\left(t\right)=\lambda \left(t\right)\left({X}_{best}^{d}\left(t\right)-{X}_{i}^{d}\left(t\right)\right)$$

where, $\lambda \left(t\right)$ denotes the Lagrangian multiplier and is defined as:

75 $$\lambda \left(t\right)=\beta {e}^{-\frac{20t}{T}}$$

$\beta$ signifies the multiplier weight.

#### Mathematical representation of atomic motion

The acceleration of the ${i}^{th}$ atom at time *t* can be computed using the interaction force and the geometric constraint as shown in Eq. (76).

76 $${a}_{i}^{d}\left(t\right)=\frac{{F}_{i}^{d}(t)}{{m}_{i}^{d}(t)}+\frac{{G}_{i}^{d}(t)}{{m}_{i}^{d}(t)}$$

where, ${m}_{i}^{d}$ is the mass of *i*th atom at time *t* in *d*th dimension, ${F}_{i}^{d}$ is the interactive force on *i*th atom, ${G}_{i}^{d}$ symbolises the constraint force on *i*th atom at time *t*. The mass of *i*th atom at time t is defined as:

77 $${M}_{i}\left(t\right)={exp}^{\frac{{Fit}_{i}\left(t\right)-{Fit}_{best}\left(t\right)}{{Fit}_{worst\left(t\right)}-{Fit}_{best}\left(t\right)}}$$

78 $${m}_{i}\left(t\right)=\frac{{M}_{i}\left(t\right)}{\sum_{j=1}^{N}{M}_{j}\left(t\right)}$$

${Fit}_{best}\left(t\right)$ and ${Fit}_{worst}\left(t\right)$ is the fitness of the search agents with best and the worst fitness value at the t^th^ iteration and are defined as:

79 $${Fit}_{best}\left(t\right)=\underset{i\in \left\{\mathrm{1,2},\dots \dots ..,N\right\}}{\mathrm{min}}{Fit}_{i}\left(t\right)$$

80 $${Fit}_{worst}\left(t\right)=\underset{i\in \left\{\mathrm{1,2},\dots ..,N\right\}}{\mathrm{max}}{Fit}_{i}\left(t\right)$$

where, ${Fit}_{i}\left(t\right)$ is the fitness of the ith agent at the tth iteration. The velocity of search agents are updated as:

81 $${v}_{i}^{d}\left(t+1\right)={rand}_{i}^{d}{v}_{i}^{d}\left(t\right)+{a}_{i}^{d}\left(t\right)$$

where, ${v}_{i}^{d}\left(t+1\right)$ is the velocity of ith search agent in the dth dimension at (t + 1)^th^ time and ${v}_{i}^{d}\left(t\right)$ is the velocity of ith search agent in the dth dimension at tth time. The position of ith search agent in dth dimension is updated as:

82 $${X}_{i}^{d}\left(t+1\right)={X}_{i}^{d}\left(t\right)+{v}_{i}^{d}\left(t+1\right)$$

### Coot bird optimization (CBO)

**Figure 15:**
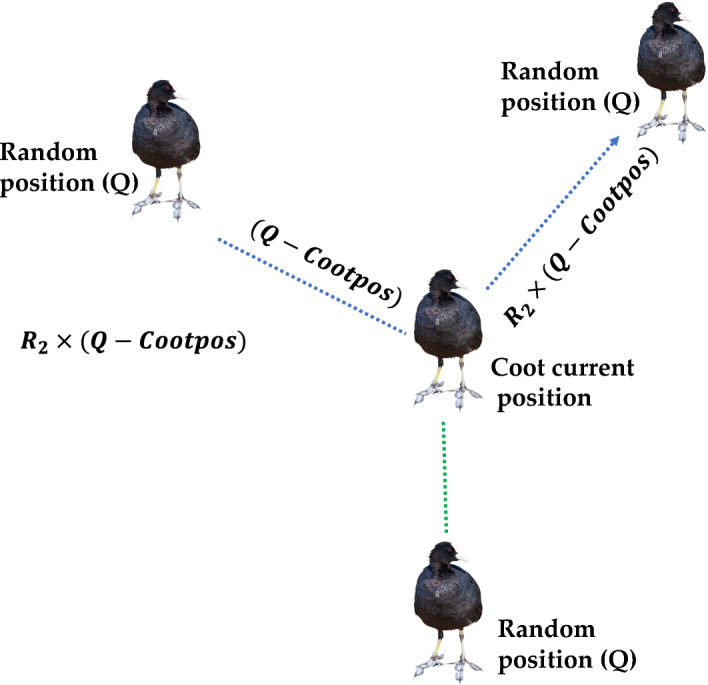
Searching mechanism of coot birds.

**Figure 16:**
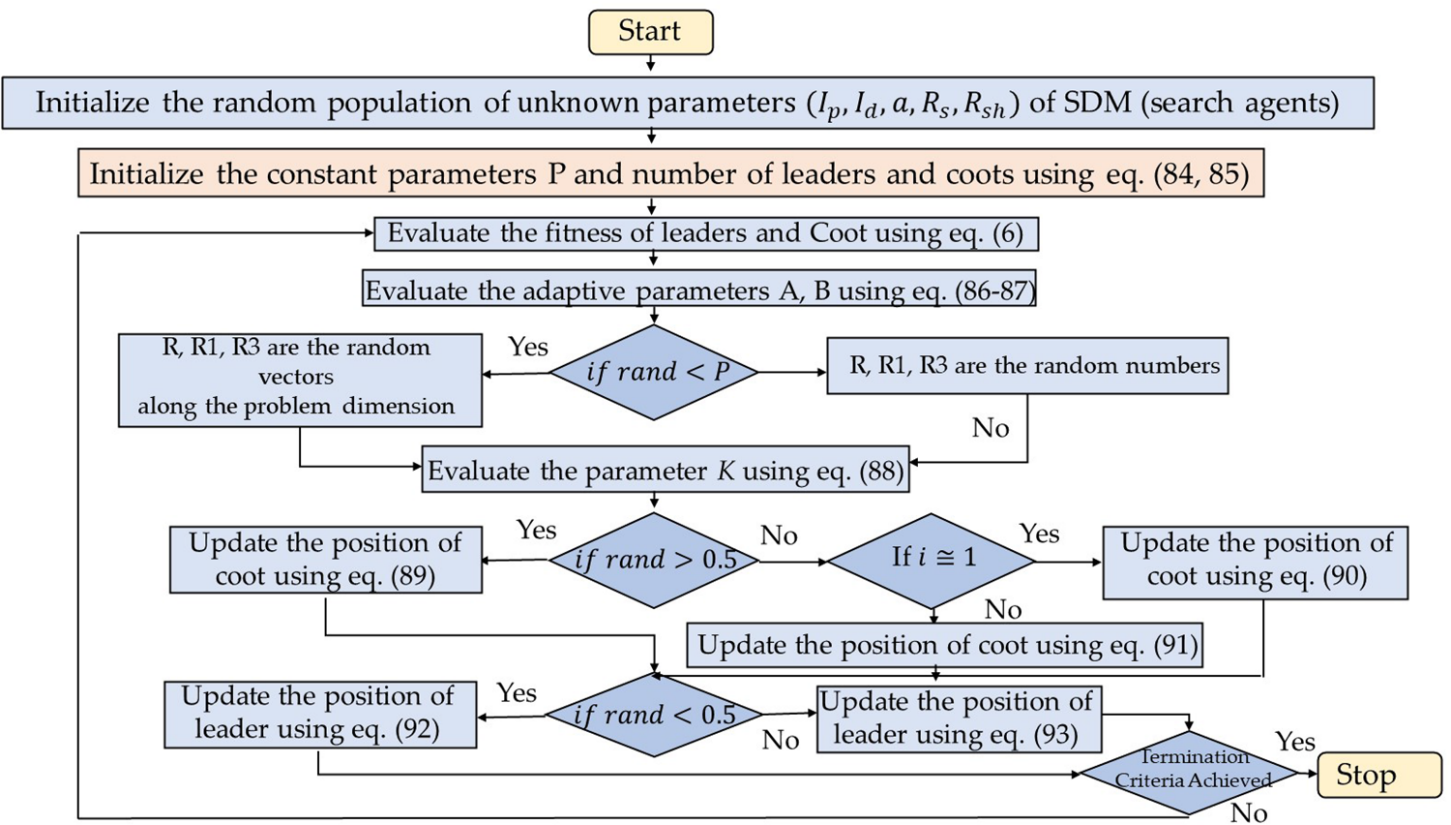
Process flow diagram of CBO algorithm.

Coot bird optimization (CBO) algorithm is a swarm intelligence algorithm proposed by Naruei and Keynia^45^ in 2021. COOT are medium size water birds that belong to the rail family, Rallidae. These birds have frontal shields on the forehead and dark red eyes with colored bills. Coots have rounded wings with physically weak fliers but have long lobed toes and strong legs which help them to run on uneven surfaces. CBO algorithm emulates three different modes of movement of Coots on the water surface that are irregular movement, regular movement and chain movement. In the third phase of chain movement coots move behind the leading leaders in the form of chains so as to quickly find the food sources. CBO algorithm mathematically models these three Coots movement so as to find the global optimal solution of any optimization problem. Figures 15 and 16 depict the CBO algorithm's search behavior and its process flow diagram. The mathematical representation of CBO algorithm is provided as follows:

*Step 1* Initialize the population of Coots (search agents) randomly in the defined space:

83 $$\overrightarrow{X}=\left({\overrightarrow{x}}_{1}, {\overrightarrow{x}}_{2},\dots \dots \dots \dots \dots \dots \dots {\overrightarrow{x}}_{n}\right)$$

where, n signifies the space dimension.

*Step 2* Initialize the constant parameters P and divide the population into leaders and coots as

84 $$NL=\left[N\times P\right]$$

where, NL, N is the number of leaders and population size. The number of Coots are

85 $${N}_{Coots}=\left[N-Leaders\right]$$

*Step 3* Evaluate the cost of all Coots, depending on the problem (minimization or maximization), the location of the finest Coot (leader) signifies global optimal solution (gBest).

*Step 4* Evaluate the adaptive parameter *A, B* as follows:

86 $$A=1-L\times \left(\frac{1}{Iter}\right)$$

87 $$\mathrm{B}=2-L\times \left(\frac{1}{Iter}\right)$$

where, *L* is the current iteration and Iter is the maximum iteration.

*Step 5* Find out another constant parameters R, R1, R3 as:

$$if\, rand<P$$

R, R1, R3 are the random vectors along the problem dimension.else

R, R1, R3 are the random numbers.

*Step 6* Evaluate the constant parameter *K* as follows:

88 $$K=1+\left(iMODNL\right)$$

where, *i* is the index number of Coot and K is the index number of leader.

*Step 7* Update the position of Coots as follows:

$$if\, rand>0.5$$

89 $$CootPos\left(i\right)=leaderPos\left(K\right)+2*R1*\mathrm{cos}\left(2R\pi \right)*\left(LeaderPos\left(K\right)-CootPos(i)\right)$$

else

$$if\, rand<0.5,i\cong 1$$

90 $$CootPos\left(i\right)=0.5*\left(CootPos\left(i-1\right)+CootPos(i)\right)$$

else

91 $$CootPos\left(i\right)=CootPos\left(i\right)+A*R2*\left(Q-CootPos(i)\right)$$

*Step 8* If the position of Coot is better than leader position then exchange Coot and leader position.

*Step 9* Update the leader position as per the following equations:

$$if\, rand<0.5$$

92 $$LeaderPos\left(i\right)=B*R3*\mathrm{cos}\left(2R\pi \right)*\left(gBest-LeaderPos\left(i\right)\right)+gBest$$

else

93 $$LeaderPos\left(i\right)=B*R3*\mathrm{cos}\left(2R\pi \right)*\left(gBest-LeaderPos\left(i\right)\right)-gBest$$

*Step 10* If the position of leader is better than gBest then exchange leader and gBest position.

*Step 11* Reinitialize the position of Coots that go beyond the defined space.

*Step 12* The algorithm terminates when the minimal error or maximum number of iterations is reached. Alternatively, resume steps (4)–(11).

*Step 13* The location of Coots with respect to gBest signifies the global optimal solution.

## Results and discussion

All MAs described in Section “Estimation of solar cell/module” are employed in this section to tackle the solar cell or PV module parameter estimation problem. Four different case studies known as R.T.C. France solar cell, Solarex MSX-60 (polycrystalline), LSM 20 (monocrystalline), and SS2018P (polycrystalline) are considered for the performance comparisons of eight selected MAs in solving various types of solar cell or PV module parameter estimation problems. Particularly, the experimental values of current and voltage for R.T.C. France solar cell are taken from (Table 10, appendix)^48^ at standard temperature condition i.e., 1000 $\mathrm{W}/{\mathrm{m}}^{2}$ at 33 ℃. For the LSM 20 PV panel that is made up of 20 monocrystalline solar cells connected in a series arrangement, the experimental measurements for current and voltage are obtained from (Table 11, appendix)^49^ and determined at the environmental condition of 360 $\mathrm{W}/{\mathrm{m}}^{2}$ and 24 °C. The SS2018P PV module involves 36 polycrystalline solar cells arranged in series, where its current and voltage readings were evaluated at the room temperature at 25 °C under different irradiation levels of 720 $\mathrm{W}/{\mathrm{m}}^{2}$, 870 $\mathrm{W}/{\mathrm{m}}^{2}$, and 1000 $\mathrm{W}/{\mathrm{m}}^{2}$. The current and voltage of SS2018P PV module are also tested at a varied resistive load (0.1–250 Ω, 2 A) (Table 13, appendix)^50^. Solarex MSX-60 polycrystalline PV module is made up of 36 solar cells connected in series, where its current and voltage are measured at a constant temperature of 25 °C under a constant irradiance of 1000 W/m^2^ (Table 12, appendix). All selected MAs are implemented and simulated using the MATLAB 2021a platform installed in a laptop with the specifications of Intel ® core ™ i7-HQ CPU, 2.4 GHz, 16 GB RAM. Table 3 displays the parameter settings adopted by all eight MAs when solving the four case studies of solar cell or PV module parameter estimation problems.

**Table 3:** Parameter settings of each algorithm.

Algorithms	Parameter	Value
SHO	Number of iterations	50,000
	Search agents	30
	Control parameter ($\overrightarrow{h}$)	[5, 0]
	$\overrightarrow{M}$ Constant	[0.5, 1]
STO	Number of iterations	50,000
	Control parameter (${C}_{f}$)	2
	Constant (*u* and *v*)	1
	Population size	30
AO	Number of iterations	50,000
	Search agents	30
	Constant (ω)	0.005
	Constant (*U*)	0.00565
	Adjustment parameter (*α, δ*)	0.1
HHO	Number of iterations	50,000
	Search agents	30
	Constant (β)	1.5
WHO	Number of iterations	50,000
	Search agents	30
	Crossover percentage (PC)	0.13
	Stallions’ percentage (PS)	0.2
AOA	Number of iterations	50,000
	Search agents	30
	α	5
	μ	0.5
ASO	Number of iterations	50,000
	Search agents	30
	Depth weight	50
	Multiplier weight	0.2
CBO	Number of iterations	50,000
	Search agents	30

**Figure 17:**
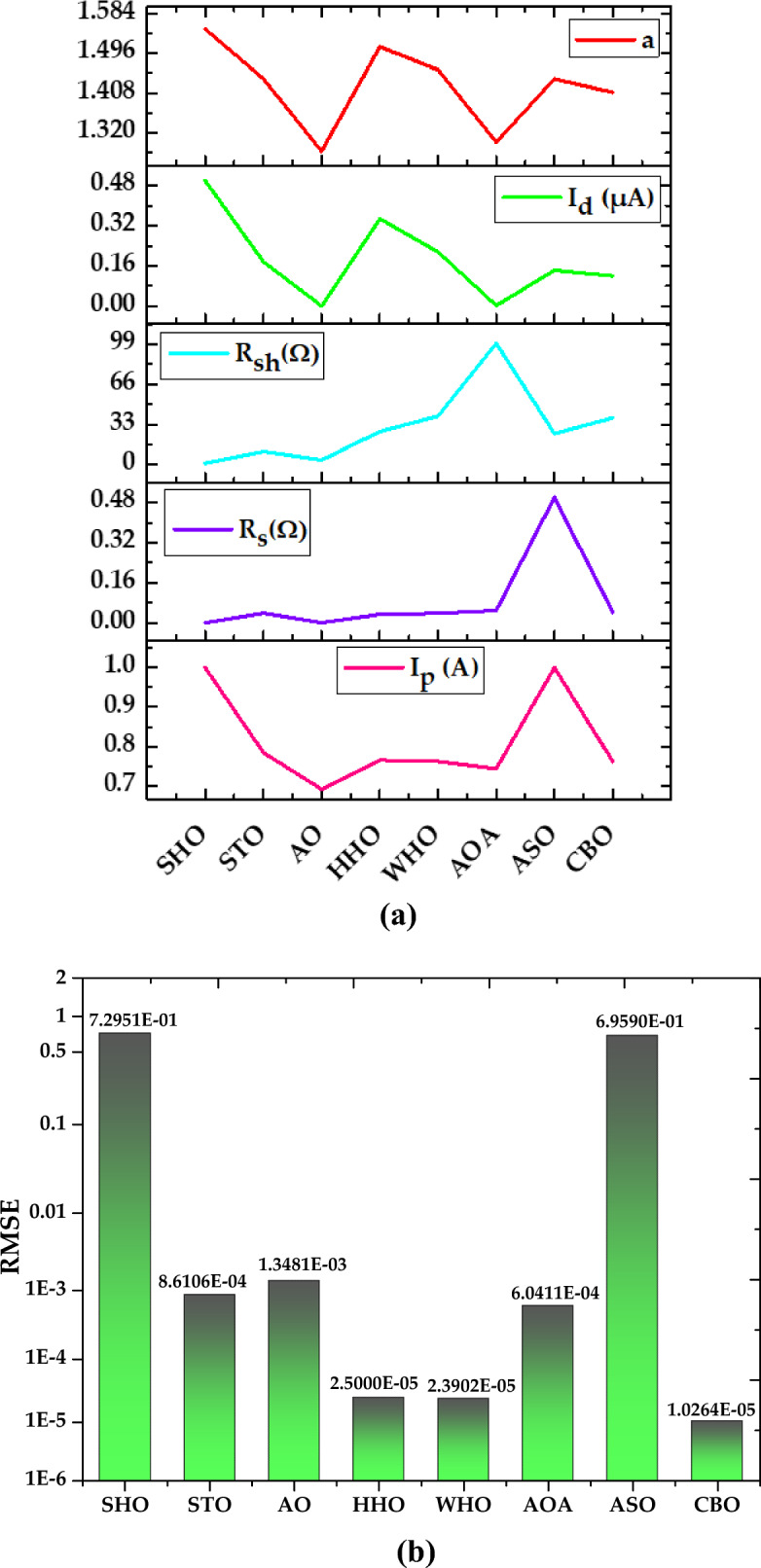
Simulation results for R.T.C. France solar cell (**a**) optimized value of all parameters (**b**) RMSE value.

**Figure 18:**
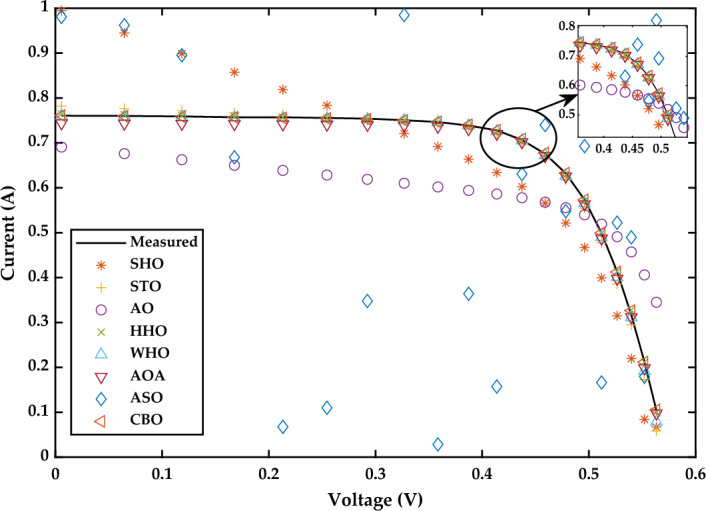
I–V Characteristics curve of simulated and experimental values by different optimization techniques for single diode model of R.T.C. France solar cell.

**Figure 19:**
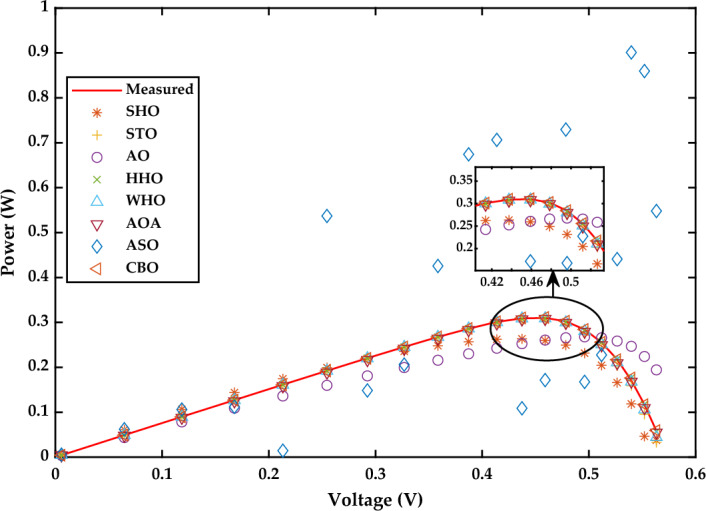
P–V Characteristics curve of simulated and experimental values by different optimization techniques for single diode model of R.T.C. France solar cell.

*Case Study 1* In this case study, the performances of all selected MAs to estimate the unknown parameters of SDM that represents the R.T.C. France solar cell operated under standard temperature condition are evaluated. The optimal values of five parameters (*I*_*p*_*, I*_*sd*_*, a, R*_*s*_*, R*_*sh*_) produced by all MAs to represent the SDM of R.T.C. France solar cell are presented in Fig. 17a and b. Meanwhile, the experimental and simulated characteristics curves of current–voltage (I–V) and power–voltage (P–V) for R.T.C. France solar cell are illustrated in Figs. 18 and 19, respectively.

**Figure 20:**
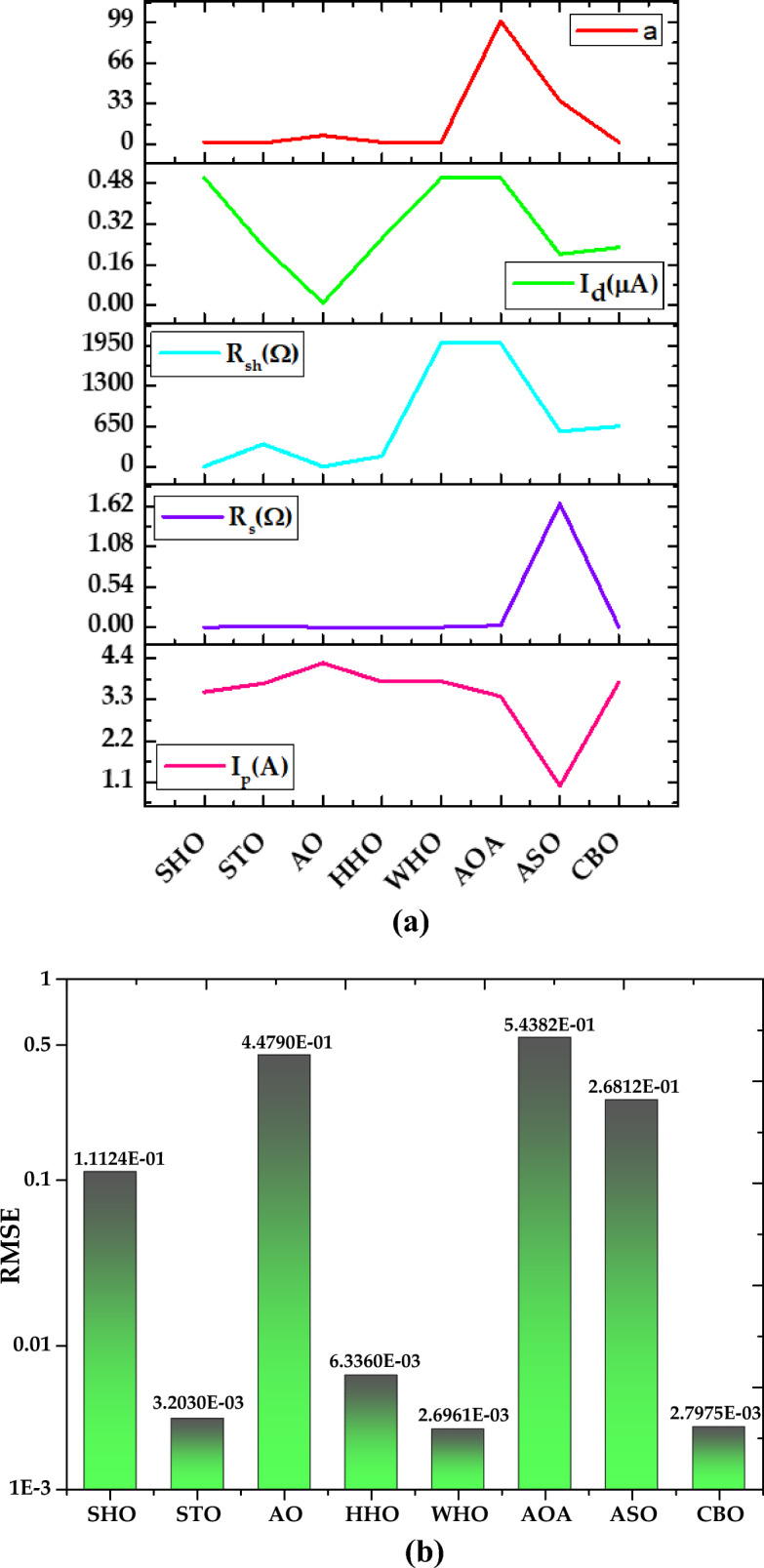
Simulation results for Solarex MSX-60 PV module (1000 W/m^2^, 25 °C) (**a**) optimized value of all parameters (**b**) RMSE value.

It is evident from Fig. 17b that CBO produces the best estimation of SDM parameter to represent the R.T.C. France solar cell with minimum RMSE value of 1.0264E−05, followed by WHO and HHO to produce the RMSE with second best and third best values of 2.3902E−05 and 2.5000E−05, respectively. In contrast, both of SHO and ASO are reported to produce the worst and second-worst RMSE values of 7.2951E−01 and 6.9590E−01, respectively. The sluggish search rate of poor solution accuracy demonstrated by ASO can be justified by its search mechanisms that solely rely on the atom force motion paradigm in molecular dynamics. On the other hand, the search mechanisms of SHO are proven not robust enough to handle the complex search space with nonlinear and multimodal characteristics, therefore it tends to suffer with premature convergence issue when solving the parameter estimation problem of the R.T.C. France solar cell (Fig. 20). A detailed comparison of eight selected algorithms with the techniques reported in the literature is illustrated in Table 6 (Appendix).

**Figure 21:**
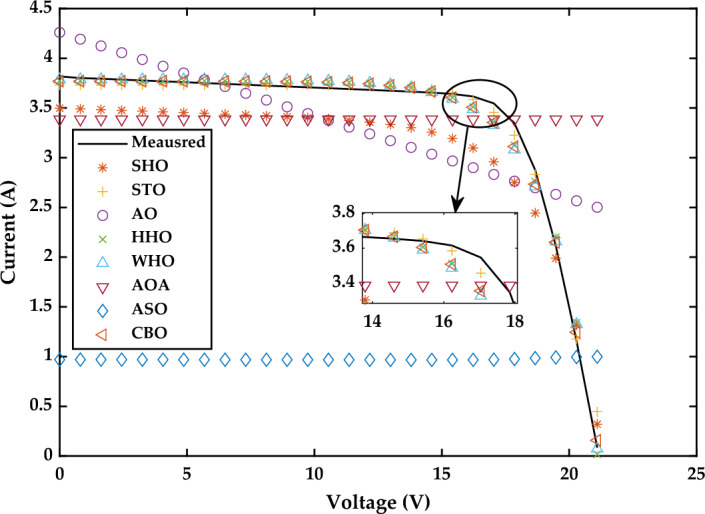
I–V Characteristics curve of simulated and experimental values by different optimization techniques for single diode model of Solarex MSX-60 PV module at STC.

**Figure 22:**
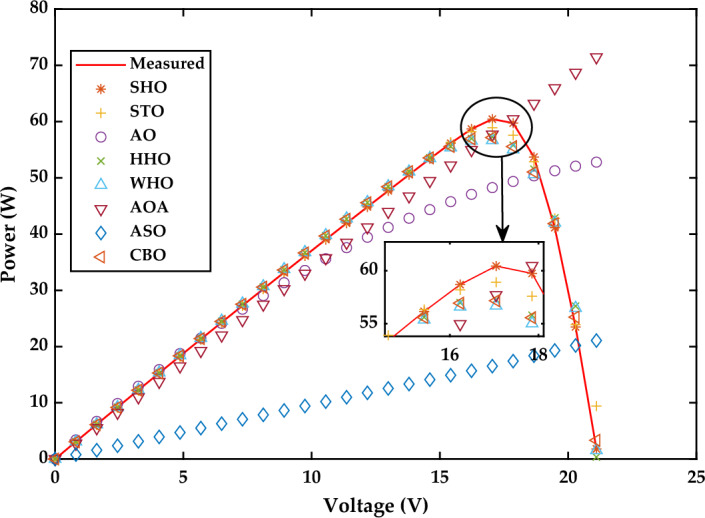
P–V Characteristics curve of simulated and experimental values by different optimization techniques for single diode model of Solarex MSX-60 PV module at STC.

*Case Study 2* The performance of all algorithms is examined in this case study for a multi-crystalline Solarex MSX-60 PV module at constant temperature of 25 °C and irradiance value of 1000 W/m^2^ using the SDM. The current–voltage and power voltage characteristics curves for Solarex MSX-60 PV module have been redrawn, as shown in Figs. 21 and 22, respectively.

Figure 20a displays the estimated values of all five parameters of SDM. According to the results indicated in Fig. 20b, WHO performs the best, followed by CBO, STO and HHO. WHO algorithm produces the RMSE value as 2.6961E−03. The RMSE values obtained by CBO, STO and HHO as 2.7975E−03, 3.2030E−03, and 6.336E−03, respectively. The good performance of WHO technique is due to a proper stability between exploration and exploitation phase. The worst results are obtained by SHO, ASO, AO and AOA, respectively. A detailed comparison of eight selected algorithms with the techniques reported in the literature is illustrated in Table 7 (Appendix).

**Figure 23:**
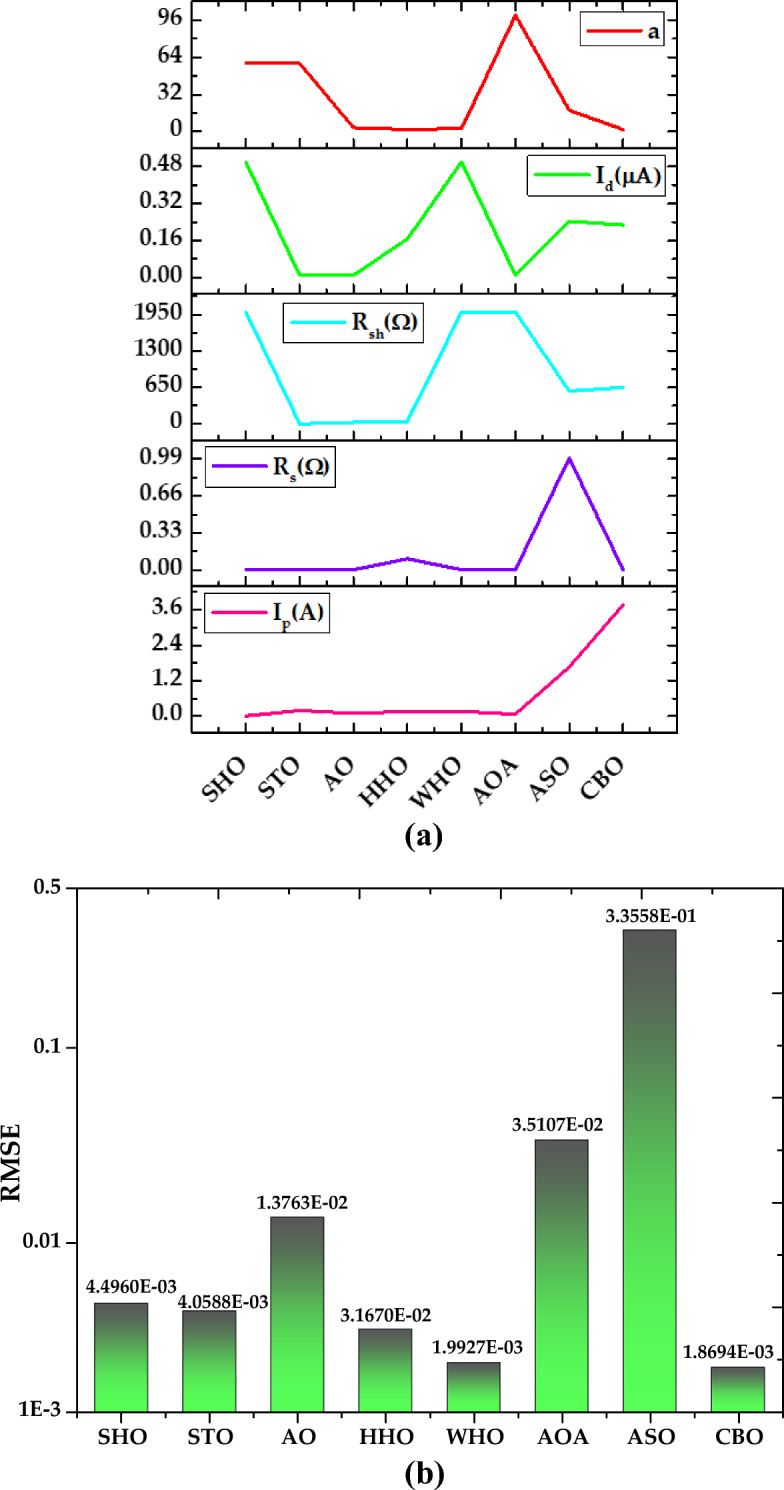
Simulation results for Leibold PV module (LSM 20) (360 W/m^2^, 24 °C) (**a**) optimized value of all parameters (**b**) RMSE value.

**Figure 24:**
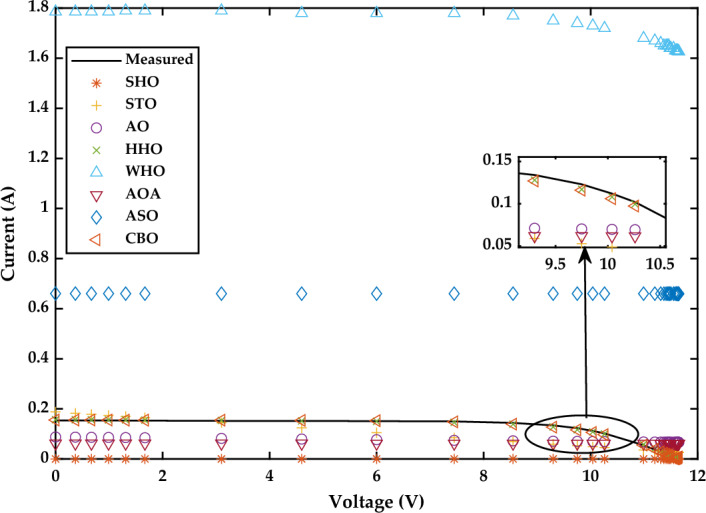
I–V Characteristics curve of simulated and experimental values by different optimization techniques for single diode model of Leibold solar module (LSM 20).

*Case Study 3* In this case study, the performance of all the algorithms is assessed for monocrystalline LSM 20 PV module at low irradiance of 360 W/m^2^ and temperature of 24 °C, by implementing the SDM. The optimal values of all five parameters for SDM of the LSM 20 PV module are illustrated in Fig. 23a. The characteristics curve of current–voltage for LSM 20 PV module is redrawn which is clearly depicted in Fig. 24.

Based on the findings in Fig. 23b, CBO performs best, followed by WHO, HHO, STO, and SHO. The CBO algorithm yields the best RMSE value of 1.8694E−03. WHO, HHO, STO, and SHO obtained RMSE values of 1.9927E−03, 3.167E−03, 4.0588E−03, and 4.4960E−03, respectively. The CBO algorithm's good efficiency is attributed to the prevention of early convergence under small irradiance values. AO, AOA, and ASO acquire the worst RMSE values of 1.3763E−02, 3.5107E−02, and 3.3558E−01, respectively. A detailed comparison of eight selected algorithms with the techniques reported in the literature is illustrated in Table 8 (Appendix).

**Figure 25:**
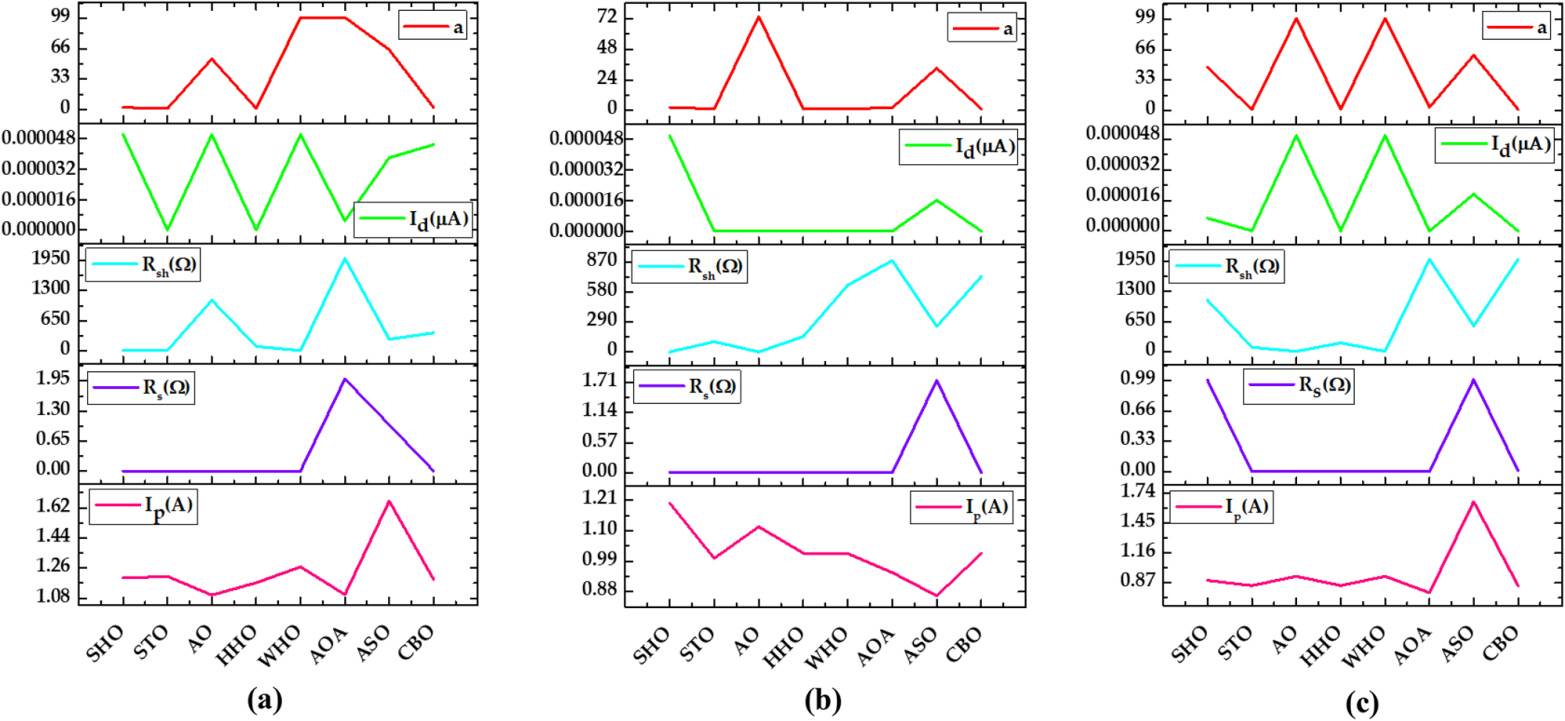
Simulation results for SS2018 PV module (**a**) optimized value of all parameters at 1000 W/m^2^ (**b**) optimized value of all parameters at 870 W/m^2^ (**c**) optimized value of all parameters at 720 W/m^2^.

**Figure 26:**
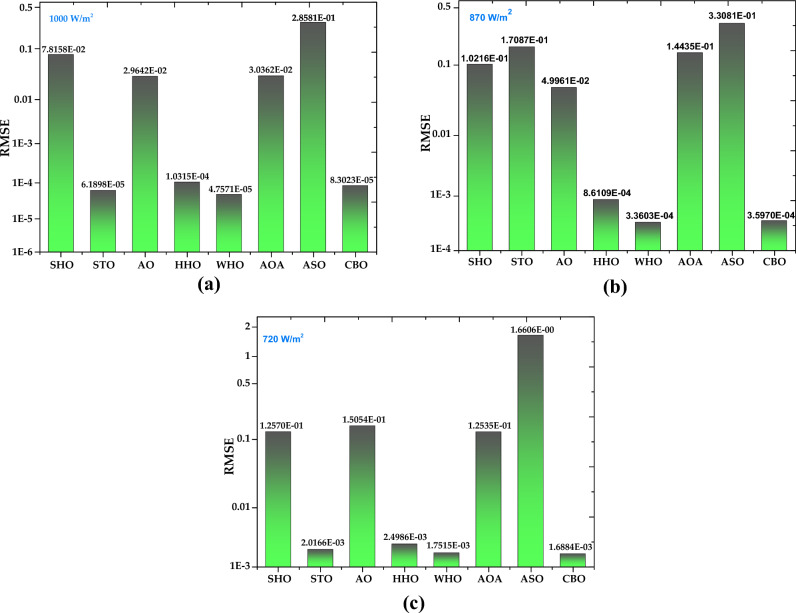
RMSE value for SS2018 PV module at (**a**) 1000 W/m^2^ (**b**) 870 W/m^2^ (**c**) 720 W/m^2^.

**Figure 27:**
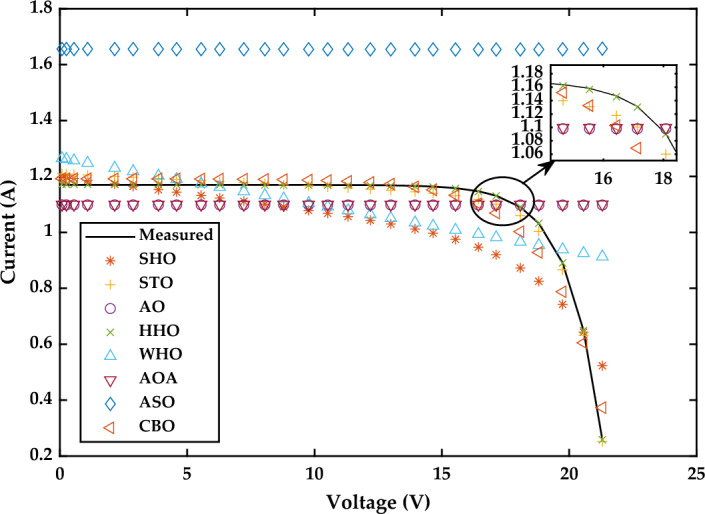
I–V Characteristics curve of simulated and experimental values by different optimization techniques for single diode model of SS2018P PV module at 1000 W/m^2^.

**Figure 28:**
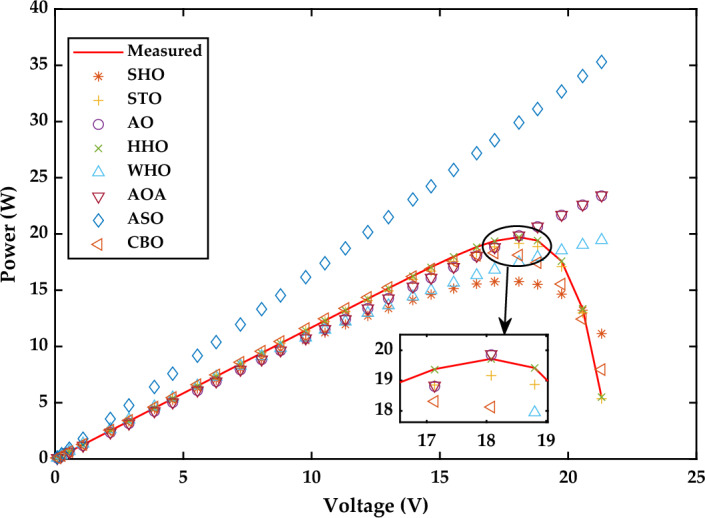
P–V Characteristics curve of simulated and experimental values by different optimization techniques for single diode model of SS2018P PV module at 1000 W/m^2^.

*Case Study 4* In this case study, the SDM is used to evaluate the performance of all methods for a polycrystalline SS2018 PV module at a constant temperature of 25 °C and different irradiance levels of 1000 W/m^2^, 870 W/m^2^, and 720 W/m^2^. Figure 25a–c depicts the optimal value of all five parameters for SDM of the SS2018 PV module at irradiance levels of 1000 W/m^2^, 870 W/m^2^, and 720 W/m^2^, respectively (Fig. 26). The current–voltage and power–voltage characteristics curves for the SS2018 PV module at 1000 W/m^2^ are redrawn, as shown in Figs. 27 and 28.

Based on the findings in Fig. 26, WHO performs best, followed by STO and CBO at 1000 W/m^2^. Similarly at 870 W/m^2^ WHO algorithm yields the best RMSE, followed by CBO and HHO. While at 720 W/m^2^, CBO algorithm produces the best RMSE, followed by WHO, STO, and HHO. A detailed comparison of eight selected algorithms with the techniques reported in the literature is illustrated in Table 9 (Appendix).

### Convergence analysis

**Figure 29:**
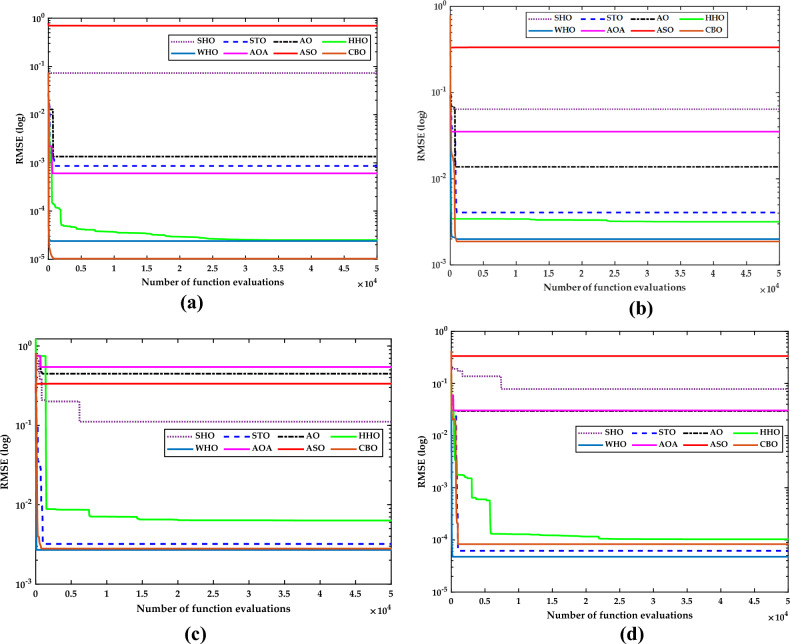
Convergence plot for (**a**) R.T.C. France solar cell (**b**) LSM20 PV module (**c**) Solarex MSX-60 PV module (**d**) SS2018 PV module.

Figure 29 describes the convergence curves of SDM for all the four-case studies to estimate the computational competence of all the selected metaheuristic techniques. It is very clear from Fig. 29 (a) that for the case of the R.T.C. France solar cell, the CBO technique obtains an accurate solution for the same number of function evaluations (i.e.,50,000) with a minimum computational time of 0.15 s as compared to other metaheuristic algorithms. CBO’s best performance is due to good exploration. Similarly, the best performance of the CBO algorithm can be seen in the instance of the thin film LSM20 PV module. The WHO and HHO algorithms generate the second-best results as compared to the CBO algorithm. The worst values of RMSE come from AO, SHO, STO, AOA, and ASO. This is because these algorithms have a problem `called premature convergence, which is caused by uneven exploitation and exploration.

In the case of the Solarex MSX-60 PV module and the SS2018 PV module, as shown in Fig. 29c and d, WHO gives the best optimized results in terms of RMSE. However, WHO algorithm lacks in terms of computational speed as compared to CBO algorithms. The CBO and STO algorithms generate the second-best results as compared to the WHO algorithm. The worst values of RMSE come from AO, SHO, AOA, and ASO. This is because these algorithms get stuck in local minima, which is caused by poor exploration of the solution space.

### Robustness and statistical investigation

This subsection offers statistical judgments based on mean, minimum, maximum, and standard deviation in terms of RMSE for all previously described procedures, as well as a comparative study with the reliability of the different approaches in a total of thirty runs, as shown in Table 4. The mean of the RMSE is computed to assess the precision of the procedures, and the standard deviation is calculated to assess the dependability of the chosen parameter estimate method.

**Table 4:** Statistical outcomes of RMSE of different algorithms for all three models.

PV module	Algorithm	RMSE				
		Min	Mean	Max	SD	Rank-Sum
R.T.C. France solar cell	SHO	7.2951E−01	7.2951E−01	7.2951E−01	3.2600E−03	** − **
	STO	8.6106E−04	9.4761E−04	2.6964E−02	1.1722E−03	** − **
	AO	1.3481E−03	1.5153E−03	1.2788E−02	1.3731E−03	** − **
	HHO	2.5000E−05	8.8800E−05	2.4324E−02	9.1477E−04	**≈**
	WHO	2.3902E−05	3.0000E−05	2.8170E−02	3.1727E−04	**≈**
	AOA	6.0411E−04	6.2354E−04	1.0885E−02	5.4195E−04	** − **
	ASO	6.9590E−01	6.9697E−01	7.8716E−01	1.7870E−03	** − **
	CBO	**1.0264E−05**	**1.8600E−05**	7.4254E−02	**2.1891E−04**	+
LSM20 PV module	SHO	4.4960E−03	6.4182E−02	1.2059E−01	**4.5861E−04**	−
	STO	4.0588E−03	4.6891E−03	8.9984E−02	5.1076E−03	−
	AO	1.3763E−02	1.3763E−02	2.6624E−01	1.0162E−02	−
	HHO	3.1670E−03	3.3336E−03	5.0246E−01	2.7905E−03	−
	WHO	1.9927E−03	2.2954E−03	9.5377E−02	6.8037E−04	≈
	AOA	3.5107E−02	3.5107E−02	7.0219E−02	1.5608E−03	−
	ASO	3.3558E−01	3.3551E−01	2.6751E−01	1.0136E−02	−
	CBO	**1.8694E−03**	**2.0604E−03**	7.9177E−01	5.4145E−04	≈
Solarex MSX-60 PV module	SHO	1.1124E−01	1.2816E−01	1.1078E−00	6.3931E−02	−
	STO	3.2030E−03	5.3431E−03	7.6117E−01	3.3049E−02	−
	AO	4.4790E−01	4.5228E−01	7.7609E−01	3.5513E−02	−
	HHO	6.3360E−03	2.8033E−02	1.2292E−00	1.2387E−02	−
	WHO	**2.6961E−03**	**3.0963E−03**	8.2015E−01	**7.9895E−04**	+
	AOA	5.4382E−01	5.4668E−01	7.5013E−01	2.4043E−02	−
	ASO	2.6812E−01	3.3548E−01	2.5471E−01	1.6006E−02	−
	CBO	2.7975E−03	4.1348E−02	8.2282E−01	8.7457E−04	≈
SS2018P PV module	SHO	7.8158E−02	8.8517E−02	2.1059E−01	2.6029E−02	−
	STO	6.1898E−05	6.1650E−05	1.3300E−02	8.9852E−04	−
	AO	2.9642E−02	2.9642E−02	3.0433E−02	9.1209E−03	−
	HHO	1.0315E−04	4.8579E−04	1.5552E−01	2.8096E−03	−
	WHO	**4.7571E−05**	**3.9300E−06**	3.0389E−02	**5.5913E−04**	+
	AOA	3.0362E−02	3.0554E−02	5.9491E−02	2.3538E−03	−
	ASO	2.8581E−01	3.3551E−01	2.9293E−01	4.4798E−03	−
	CBO	8.3023E−05	4.0300E−04	4.1757E−01	5.8557E−04	≈

Significant values are in bold.

The result of the statistical study shows that the CBO technique outperforms other optimization techniques for both case studies R.T.C. France Solar cell and LSM20 PV module which validates its superior exploration and exploitation capability. On the other hand, WHO technique provides best results for the case of Solarex MSX-60 PV module and SS2018 PV module because of low standard deviation and high accuracy. HHO gives the third best performance for case study 1 and 3 as it suffers from poor population diversity. According to NFL^29^, it is not necessary that if one algorithm gives superior performance on a specific problem, it may perform the same on other problems. There is no one-size-fits-all solution to problem-solving, and the most effective approach will depend on the specific context and constraints of the problem at hand.

The Wilcoxon rank-sum test is a nonparametric metric used to compare the results of two approaches. It is employed to examine the population distributions of two independent samples to see if they are equal. This test quantifies the relevance of a variation between two samples and does not assume that the data is normally distributed, making it a useful alternative when normality assumptions cannot be met. The Wilcoxon rank-sum test works by first ranking the combined data from both samples, then determining the sum of ranks for each sample. The null hypothesis denotes that the rankings of the comparison methodologies' results are not notably different. The alternative hypothesis looks into whether the outcomes of the comparative approach may be described by rank. The Wilcoxon rank-sum was calculated with a significance threshold of 5%. The sign “ + ” indicates that the compared algorithm won the other algorithm significantly, the sign “≈” indicates that the implemented algorithm performed similarly to the other algorithm, and the sign “ − ” indicates that the employed algorithm performed poorly in comparison to the other algorithm.

**Figure 30:**
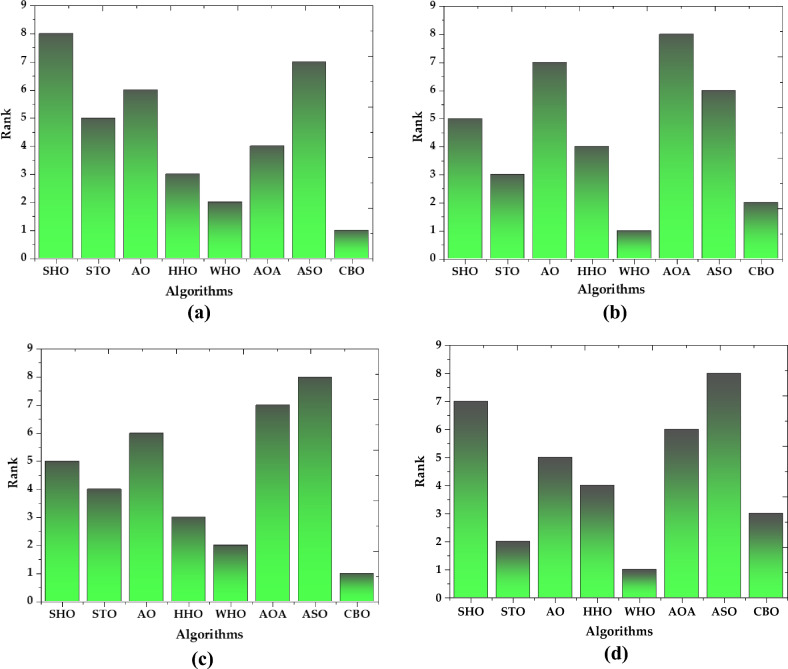
Friedman mean rank of all algorithms for (**a**) R.T.C. France solar cell (**b**) Solarex MSX-60 PV module (**c**) LSM20 PV module (**d**) SS2018 PV module.

In addition to normal statistical analysis, such as best, mean, worst, and standard deviation, the Friedman rank test^51^ is used to establish the significance of the data. It is often used in the analysis of repeated-measures designs in which multiple observations are made on the same subjects under different conditions. The test works by first transforming the data into ranks, and then summing the ranks for each subject across the conditions. The Friedman rank test is appropriate for continuous or ordinal data, and when the assumptions of normality and equal variances are not met. This non-parametric test is also used to rank the algorithms for each studied PV module. The null hypothesis (p-value 5%) in the Friedman test indicates that there is no discernible difference between the compared methods. The contrary hypothesis denotes a significant variance between the compared methods throughout all 30 runs. Each algorithm is ranked in this test depending on its performance. The best algorithms are determined by small ranks. The Friedman rank test findings at a 95% confidence level are shown in Fig. 30. According to Fig. 30, for the case of R.T.C. France solar cell, CBO is having the best performance followed by WHO, HHO, AOA, STO, AO, ASO, and SHO. While in the case of Solarex MSX-60 PV module, the best performance is given by WHO algorithm. In the case of LSM 20 PV module CBO algorithm shows its supremacy as compared to other algorithms. In the instance of the SS2018 PV module, the WHO algorithm outperforms all other algorithms.

**Figure 31:**
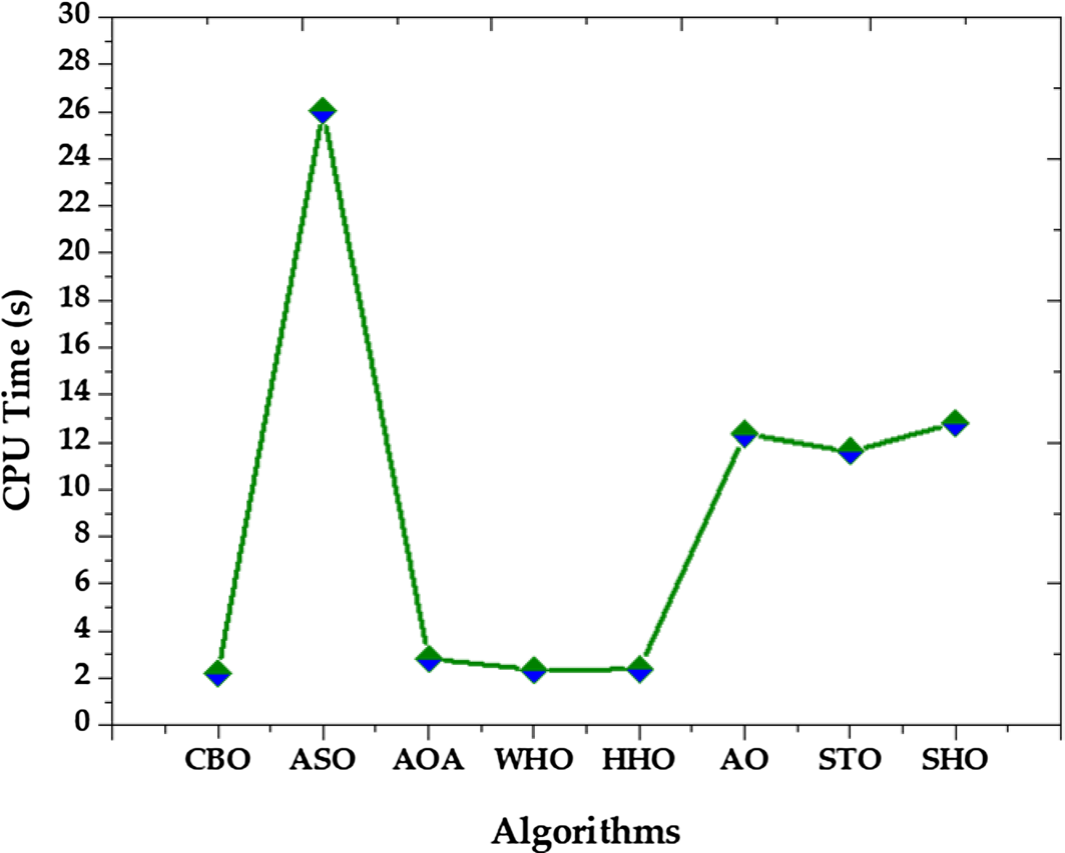
Comparison of computation time for all algorithms.

The average execution time of each algorithm on all four PV models is computed and provided in Fig. 31 in order to evaluate the efficiency of all metaheuristic techniques implemented in this research study. Metaheuristic algorithms take a certain amount of time to run based on a number of factors, such as the size and complexity of the problem, the convergence criteria, and how good the first solution is. It is very clear from Fig. 31 that the minimum average execution time is taken by the CBO technique as 1.19 s. while AOA, WHO, and HHO have more or less the same average execution time. The ASO technique takes a long time to execute, 26.05 s.

### Qualitative analysis of algorithms

This subsection discusses the metaheuristic methods presented in Section “Estimation of solar cell/module” for parameter estimation of solar cells/modules based on key performance indices which includes computational complexity, convergence speed, utilization of memory of previous states, search methodology, merits and demerits. Table 5 shows an evaluation of all the algorithms.

**Table 5:** Qualitative evaluation of recent metaheuristic methods for parameter optimization of solar cell/module.

Parameters	SHO	STO	AO	HHO	WHO	AOA	ASO	CBO
Computational complexity	$O\left(k\times T\times D\times N\right)$	$O\left(N\times T\times D\right)$	$O\left(N\times (T\times D+1\right))$	$O(N\times (T+T\times D+1)$	$O(2\times N\times D\times T+N\times D+{N}_{foal}\times T)$	$O\left(N\times \left(T+1\right)+D\right)$	$O\left(N\times T\times (D+1\right))$	$O\left(N\times D\times \left(T+1\right)\right)$
Convergence speed	Moderate	Moderate	Low	High	High	Low	Moderate	High
Memory of prior states	No	No	No	No	Yes	No	No	No
Search methodology	Mutation and Selection	Mutation and Selection	Mutation and Selection	Mutation and Selection	Mutation, Selection and Crossover	Mutation and Selection	Mutation and Selection	Mutation and Selection
Merits	High scalability	Effectively handle multidimensional problem	Good diversity	Good for high-dimensional constrained problem	Ease of implementation	Good at exploitation	Good diversity	Good at exploration
Limitations	Suffers with premature convergence	Low diversity	Stuck in local minima	Limited areas of application	Stagnation in local optimal solution	Large number of tuning parameters	Poor accuracy	Exploitation capability can be further improved

The *computational complexity* is described as the system's need for computation resources as a function of the number of search agents (population). The expected computation time and computation storage required for the solution define the resources. A metaheuristic algorithm's computational complexity is based on three regulations: solution initialization, fitness function calculation, and solution updating. *N* represents the number of solutions generated during initialization process. The total number of iterations is denoted by *T* and *D* denotes the dimension of the optimization problem. Table 5 shows that the WHO algorithm has the highest whereas the STO the least computational complexity in comparison to other algorithms.

The algorithm's convergence speed is defined as the rate at which it can locate the best solution. An efficient algorithm must have a high rate of convergence and avoid premature convergence. Premature convergence is defined as the convergence of a metaheuristic algorithm prior to achieving a global optimal solution, which is usually caused by a lack of diversity or imbalance between the searching stages. As observed from Table 5 HHO, WHO and CBO algorithm have fast rate of convergence while AO and AOA have the slow rate of convergence.

The *memory of prior states* influences the number of storage resources utilized during the execution of a metaheuristic algorithm. This parameter has an insignificant impact for a small-scale optimization problem (like estimating the parameters for solar cells), but as the number of solar cells in a PV module increase, it may become an important attribute in determining the performance of the algorithm. As per the study depicted in Table 5 among all algorithms only WHO utilizes the memory of previous states, therefore shouldn’t be considered for parameter estimation of large-scale PV modules.

The particular manner by which the algorithm tackles the problem is referred to as the search methodology. Most SI-based algorithms use one of three search methodologies: mutation, selection, or crossover. Mutation is known as the technique for global exploration. The process of selecting the best solution in the search space is known as selection. Crossover increases the search space's diversity^52^. WHO algorithm discovers the solution by utilizing all three search methodologies. It, therefore, requires more memory space in comparison to all other algorithms.

## Conclusion

This paper presents an exhaustive investigation of recently developed state-of-the-art MAs for PV cell parameter estimation, with a focus on the underlying theory and experimental efficiency of each technique on four case studies based on four distinct PV cell/module technologies under wide range of irradiance and temperature levels. The properties and attributes of different MAs have been examined for PV parameter estimation of various PV module technologies under distinct environmental conditions. The proposed study evaluates the performance of prior art MAs based on key performance indices such as convergence rate, implementation complexity, accuracy along with their merits and demerits. The main outcomes of the proposed work are:

- CBO algorithm gives the best RMSE value of 1.0264E−05 for R.T.C. France solar cell under 1000W/m^2^, 1.8694E−03 for LSM 20 PV module at 360W/m^2^, and 1.6884E−03 for SS2018 PV module at 720W/m^2^, respectively.
- WHO evaluates the best RMSE value of 2.6961E−03 for Solarex MSX-60 under 1000W/m^2^ and 4.7571E−05 under 1000W/m^2^ and 3.3603E−04 870W/m^2^ for SS2018 PV module, respectively.
- ASO, AO and AOA have lower accuracy and thus result in high RMSE value for different PV module technologies.
- CBO algorithm has the highest rate of convergence for R.T.C. France at 1000W/m^2^, LSM 20 at 360W/m^2^ and SS2018 PV module at 720W/m^2^, respectively.
- WHO algorithm has the highest rate of convergence for Solarex MSX-60 under 1000W/m^2^ and SS2018 PV module under 870W/m^2^ and 1000W/m^2^, respectively.
- Algorithms like ASO, AO, STO, and SHO take large execution time due to their high computational complexity and poor trade-off between exploration and exploitation.

This study establishes that there is no one-size-fits-all MA to solve the optimization problem, and the most effective approach will depend on the specific PV cell technology and the operating condition. As compared to earlier studies on this topic, this study has substantially expanded the diversity of algorithms, simulated outcomes, and comparison of recently anticipated techniques. As a result of this assessment, improved and hybridization of discussed algorithms can be developed for various renewable energy applications. A hardware setup consisting of a low-cost microcontroller can be used to implement these metaheuristics algorithms in real time applications.

## Data Availability

The datasets used and/or analysed during the current study available from the corresponding author on reasonable request.

## Appendix

See Tables

**Table 6:** Evaluation of all compared metaheuristic algorithms with the techniques reported in literature for the SDM of R.T.C. France solar cell.

Algorithms	I_p_ (A)	R_s_ (Ω)	R_sh_ (Ω)	I_d_ (µA)	a	RMSE
SHO	1	0.001	1.18	0.5	1.55	7.2951E−01
STO	0.785	0.0394	10.998	0.177	1.44	8.6106E−04
AO	0.6923	0.001	3.99	0.001	1.28	1.3481E−03
HHO	0.7659	0.0347	27.2342	0.348	1.5108	2.5000E−05
WHO	0.7622	0.0386	40.0396	0.2161	1.46	2.3902E−05
AOA	0.7441	0.05	100	0.0031	1.3	6.0411E−04
ASO	1	0.5	25.5045	0.143	1.44	6.9590E−01
CBO	0.7619	0.042	38.5567	0.121	1.41	1.0264E−05
FODPSO^53^	0.7609	0.0364	51.9512	0.3187	1.4806	9.7486E−04
NPSOPC^54^	0.7608	0.0363	53.7583	0.3325	1.4814	9.8856E−04
CPMPSO^55^	0.7607	0.0363	53.7185	0.3230	1.4811	9.8602E−04
ABC^56^	0.7608	0.0364	54.6433	0.3251	1.4817	9.8620E−04
TLABC^57^	0.7608	0.0364	53.7164	0.3230	1.4812	9.8602E−04
BHCS^58^	0.7608	0.0364	53.7185	0.3200	1.4800	1.0000E−03
MPCOA^59^	0.7607	0.0364	54.6328	0.3366	1.4817	9.4457E−04

^6^,

**Table 7:** Evaluation of all compared metaheuristic algorithms with the techniques reported in literature for the SDM of Solarex MSX-60 PV module.

Algorithms	I_p_	I_d_ (μA)	R_s_	R_sh_	a	CPU Time (s)	RMSE
SHO	3.5	0.5	0.001	3.05	2.05	36.6	1.1124E−01
STO	3.7313	0.231	0.0049	359.7437	1.35	0.1	3.2030E−03
AO	4.271765	0.01	0.001	0.331239	7.61543	0.23	4.4790E−01
HHO	3.7741	0.263	0.001	170.6833	1.8996	10.01	6.3360E−03
WHO	3.7859	0.5	0.001	2000	2.0103	0.37	2.6961E−03
AOA	3.3865	0.5	0.0238	2000	100	11.01	5.4382E−01
ASO	1	0.201	1.6606	567.3439	35.54	93.44	2.6812E−01
CBO	3.7702	0.228	0.001	649.7869	1.87	0.15	2.7975E−03
HS^60^	3.8115	0.2265	0.2128	1976.07	1.3707	NA	1.7756E−02
COA^61^	3.81	0.1783	0.2184	2004.977	1.3514	NA	1.7050E−02
NM^61^	3.8084	0. 0005	0.3692	169.047	1.0003	NA	9.6132E−02
BC^62^	3.8080	0. 0012	0.3160	146.080	1.04	NA	4.2026E−02
A&I^63^	3.7983	0.0679	0.2510	582.728	1.28	NA	2.5025E−02
ER-WCA^60^	3.8121	0. 1399	0.2235	914.689	1.33	NA	1.6973E−02

^7^,

**Table 8:** Evaluation of all compared metaheuristic algorithms with the techniques reported in literature for the SDM of LSM 20 PV module.

Algorithms	I_p_	I_d_ (μA)	R_s_	R_sh_	a	CPU Time (s)	RMSE
SHO	0	0.5	0.001	2000	59.27	2.35	4.4960E−03
STO	0.1878	0.0101	0.0012	3.6133	58.86	0.11	4.0588E−03
AO	0.0877	0.01	0.001	27.749	3.35	0.25	1.3763E−02
HHO	0.1573	0.168	0.0998	50.183	1.96	13.14	3.1670E−03
WHO	0.1567	0.5	0.001	2000	2.77	0.36	1.9927E−03
AOA	0.062	0.01	0.001	2000	100	0.11	3.5107E−02
ASO	1.6606	0.243	1	585.0286	18.44	5.56	3.3558E−01
CBO	3.7702	0.228	0.001	649.7869	1.87	0.33	1.8694E−03
SMA^64^	0.1550	0.0001	7.2958	1545.1678	1.07	NA	7.8030E−04
ACT^49^	0.15449	0.0025	6.3944	1973.35	1.268	NA	8.3839E−04
MRFO^65^	0.1944	0.4509	0.0019	4.579	3.10	NA	8.2751E−04
ALO^66^	0.1856	0.4529	0.0012	3.784	3.63	NA	7.6603E−03

^8^,

**Table 9:** Evaluation of all compared metaheuristic algorithms with the techniques reported in literature for the SDM of SS2018PV module (at 1000 $\mathrm{W}/{\mathrm{m}}^{2}$).

Algorithms	I_p_	I_d_ (μA)	R_s_	R_sh_	a	CPU Time (s)	RMSE
SHO	1.2	0.500	0.001	2.3	2.52	8.1	7.8158E−02
STO	1.2102	0.040	0.001	6.2091	1.35	0.14	6.1898E−05
AO	1.0985	0.500	0.001	1091.972	55.06	0.67	2.9642E−02
HHO	1.1719	0.0589	0.001	92.0987	1.3757	10.85	1.0315E−04
WHO	1.2669	0.5	0.001	1.6745	100	0.36	4.7571E−05
AOA	1.1009	4.95	1.994	2000	100	0.11	3.0362E−02
ASO	1.6606	0.379	1	247.8556	65.04	3.1	2.8581E−01
CBO	1.1919	0.447	0.001	384.8235	2.32	0.15	8.3023E−05
ALO^66^	1.0849	0.3339	0.0130	1923.00	2.25	NA	1.3875E−02
SCA^67^	1.0400	0.1643	0.0831	971.14	23.51	NA	1.0735E−02
MRFO^65^	1.1964	0.3645	0.0011	1926.54	1.27	NA	4.7010E−02
WOA^68^	1.2734	0.4987	0.0063	1.6472	55.0	NA	1.6398E−01

^9^,

**Table 10:** Experimental measurement of current and voltage for R.T.C. France solar cell^35^.

Voltage (V)	Current (A)
0.0057	0.7605
0.0646	0.76
0.1185	0.759
0.1678	0.757
0.2132	0.757
0.2545	0.7555
0.2924	0.754
0.3269	0.7505
0.3585	0.7465
0.3873	0.7385
0.4137	0.728
0.4373	0.7065
0.459	0.6755
0.4784	0.632
0.496	0.573
0.5119	0.499
0.5265	0.413
0.5398	0.3165
0.5521	0.212
0.5633	0.1035

^10^,

**Table 11:** Experimental measurement of current and voltage for LSM 20 PV panel^36^.

Voltage (V)	Current (A)
0	0.154
0.37	0.154
0.67	0.154
0.99	0.154
1.31	0.153
1.67	0.153
3.1	0.152
4.6	0.152
6	0.151
7.45	0.15
8.55	0.144
9.3	0.134
9.75	0.123
10.04	0.112
10.26	0.102
10.99	0.055
11.2	0.037
11.31	0.0281
11.37	0.0227
11.41	0.019
11.44	0.016
11.46	0.0142
11.48	0.0127
11.5	0.012
11.55	0.006
11.58	0.003
11.6	0.002
11.61	0.001
11.62	0.001
11.63	0.0006
11.632	0.0003
11.633	0.0002
11.636	0.0001
11.64	0.0001
11.65	0

^11^,

**Table 12:** Experimental measurement of current and voltage for Solarex MSX-60 PV module^21^.

Voltage (V)	Current (A)
0	3.8174
0.8115	3.8015
1.623	3.7944
2.4346	3.7855
3.2461	3.7769
4.0576	3.7683
4.8692	3.7597
5.6807	3.7511
6.4923	3.7425
7.3038	3.7339
8.1153	3.7253
8.9269	3.7167
9.7384	3.7081
10.55	3.6995
11.3615	3.6909
12.173	3.6823
12.9846	3.6736
13.7961	3.6646
14.6076	3.6548
15.4192	3.6415
16.2307	3.6158
17.0423	3.5464
17.8538	3.3461
18.6653	2.8771
19.4769	2.1119
20.2884	1.1577
21.1	0.0847

^12^,

**Table 13:** Experimental measurement of current and voltage for SS2018P PV module^37^.

Voltage (V)	Current (A)	Current (A)	Current (A)
	(@ 1000 W/m^2^)	(@ 870 W/m^2^)	(@ 720 W/m^2^)
0.084479739	1.169877186	1.017777186	0.842277186
0.255876876	1.169782273	1.017682273	0.842182273
0.555022866	1.169752945	1.017652945	0.842152945
1.089628673	1.169750019	1.017650019	0.842150019
2.15290929	1.169752328	1.017652328	0.842152328
2.878081538	1.16974991	1.01764991	0.84214991
3.869691059	1.169754066	1.017654065	0.842154065
4.583330633	1.169748326	1.017648325	0.842148325
5.548299366	1.169751241	1.01765124	0.842151239
6.278013801	1.169741576	1.017641574	0.842141573
7.224307089	1.169737945	1.017637942	0.84213794
8.050157312	1.16971943	1.017619426	0.84211942
8.787816474	1.169684042	1.017584034	0.842084025
9.768922844	1.169625413	1.017525397	0.842025377
10.51810238	1.169502219	1.017402189	0.841902156
11.31670088	1.169288373	1.017188319	0.841688257
12.19017661	1.168853394	1.01675329	0.841253169
12.99476409	1.168090489	1.015990297	0.840490075
13.94578405	1.166352235	1.014251839	0.838751383
14.65561936	1.163882131	1.011781453	0.83628067
15.53478383	1.158313315	1.006211993	0.830710468
16.43301402	1.147140906	0.995038292	0.819535276
17.13242511	1.131259763	0.979155318	0.803650189
18.08015892	1.090733823	0.938624697	0.763114165
18.80655994	1.032566877	0.880451037	0.704932757
19.74233771	0.890636178	0.738503947	0.562966753
20.56285509	0.649390858	0.497230772	0.321661433
21.3013028	0	0	0

^13^.

## Abbreviations

ABCO: Artificial Bee Colony Optimization
ABSO: Artificial Bee Swarm Optimization
ADE: Adaptive Differential Evolution
AGA: Adaptive Genetic Algorithm
AIS: Artificial Immune System
ALO: Ant Lion Optimizer
ANN: Artificial Neural Network
APSO: Particle Swarm Optimization with Adaptive Inertia Weight Control
AVO: African Vultures Optimization
BBO-M: Bio-Geography Based Optimization with Mutation Strategies
BFA: Bacterial Foraging Algorithm
BMO: Bird Mating Optimization
BP-FPA: Bee Pollinator Flower Pollination Algorithm
BHCS: Biogeography-Based Heterogeneous Cuckoo Search
COA: Chaos Optimization Algorithm
CPSO: Chaos Particle Swarm Optimization
CS: Cuckoo Search
CSO: Cat Swarm Optimization
CPMPSO: Combined Perturbation Mutation PSO
DE: Differential Evolution
DEIM: Differential Evolution with Integral Mutation
ERWCA: Evaporation Rate Based Water Cycle Algorithm
FPA: Flower Pollination Algorithm
FWA: Fire Works Algorithm
FODPSO: Fractional Order Darwinian PSO
GA: Genetic Algorithm
GCPSO-NRM: Hybrid of GCPSO with Newton Raphson Method
GGHS: Grouping Based Global Harmony Search
GOFPANM: Generalized Opposition-based Flower Pollination Algorithm Nelder-Mead Simplex Method
GOTLBO: Generalized Oppositional Teaching Based Learning Optimization
GSA: Gravitational Search Algorithm
GWO: Grey Wolf Optimization
HBPFPA: Hybrid bee pollinator flower pollination algorithm
HS: Harmony Search
HTS: Hazelnut Tree Search Algorithm
IADE: Improved Adaptive DE
IALO: Improved Ant Lion Optimizer
ITLBO: Improved Teaching Learning Based Optimization
IBCPSO: PSO with Inverse Barrier Constraints
ICA: Imperialist competitive algorithm
IC-WOA: Improved Chaotic Whale Optimization Algorithm
IGHS: Innovative Global Harmony Search
IPSO: Improved Particle Swarm Optimization
ISCE: Improved Shuffled Complex Evolution Algorithm
JA: JAYA Algorithm
JADE: Joint Approximation Diagonalization of Eigen-matrices
LM: Levenberg–Marquardt
LMCOA: Lozi Map based Chaotic Optimization Algorithm
LS: Least Square
LCOA: Lozi Map Based Chaotic Optimization Algorithm
TLABC: Teaching Learning based ABC
MLBSA: Multiple learning backtracking search algorithm
MPCOA: Mutative-Scale Parallel Chaos Optimization
MRFO: Manta Ray Foraging Optimization
MSPCOA: Mutative Scale Parallel Chaos Optimization Algorithm
MSSO: Modified Simplified Swarm Optimization
NMS: Nelder-Mead Algorithm
NMS-ABC: Adaptive Nelder-Mead simplex (NMS) hybridized with the artificial bee colony- (ABC) metaheuristic Algorithm
NLSA: Nonlinear Least Square Algorithm
NR: Newton–Raphson
NPSOPC: Niche Particle Swarm Optimization in Parallel Computing
PDE: Penalty based DE
PSA: Parallel Swarm algorithm
PS: Pattern search
PSO: Particle Swarm Optimization
RADE: Repaired Adaptive Differential Evolution.
SA: Simulated Annealing
SCA: Sine Cosine Algorithm
SBMOA: Simplified Bird Mating Optimization Approach
SCE: Shuffled Complex Evolution
SSA: Salp Swarm Algorithm
STLBO: Simplified Teaching Learning Based Optimization
TLABCA: Teaching–learning based Artificial Bee Colony Algorithm
TLBO: Teaching Learning Based Optimization
TLO: Teaching learning-based optimization
TVIWAC-PSO: Particle Swarm Optimization with Time Varying Inertia Weight and Acceleration Coefficients
VC-PSO: Particle Swarm Optimization with Velocity Clamping
VIM: Villalva’s Iterative Method
WDO: Wind Driven Optimization
WOA: Whale Optimization Algorithm
